# Opinion Dynamics with Confirmation Bias

**DOI:** 10.1371/journal.pone.0099557

**Published:** 2014-07-09

**Authors:** Armen E. Allahverdyan, Aram Galstyan

**Affiliations:** 1 Department of Theoretical Physics, Yerevan Physics Institute, Yerevan, Armenia; 2 USC Information Sciences Institute, Marina del Rey, California, United States of America; Middlesex University London, United Kingdom

## Abstract

**Background:**

Confirmation bias is the tendency to acquire or evaluate new information in a way that is consistent with one's preexisting beliefs. It is omnipresent in psychology, economics, and even scientific practices. Prior theoretical research of this phenomenon has mainly focused on its economic implications possibly missing its potential connections with broader notions of cognitive science.

**Methodology/Principal Findings:**

We formulate a (non-Bayesian) model for revising subjective probabilistic opinion of a confirmationally-biased agent in the light of a persuasive opinion. The revision rule ensures that the agent does not react to persuasion that is either far from his current opinion or coincides with it. We demonstrate that the model accounts for the basic phenomenology of the social judgment theory, and allows to study various phenomena such as cognitive dissonance and boomerang effect. The model also displays the order of presentation effect–when consecutively exposed to two opinions, the preference is given to the last opinion (recency) or the first opinion (primacy) –and relates recency to confirmation bias. Finally, we study the model in the case of repeated persuasion and analyze its convergence properties.

**Conclusions:**

The standard Bayesian approach to probabilistic opinion revision is inadequate for describing the observed phenomenology of persuasion process. The simple non-Bayesian model proposed here does agree with this phenomenology and is capable of reproducing a spectrum of effects observed in psychology: primacy-recency phenomenon, boomerang effect and cognitive dissonance. We point out several limitations of the model that should motivate its future development.

## Introduction

*Confirmation bias* is the tendency to acquire or process new information in a way that confirms one's preconceptions and avoids contradiction with prior beliefs [52]. Various manifestations of this bias have been reported in cognitive psychology [5], [67], social psychology [24], [54], politics [46] and (media) economics [31], [51], [57], [73]. Recent evidence suggests that scientific practices too are susceptible to various forms of confirmation bias [12], [38], [43], [44], [52], even though the imperative of avoiding precisely this bias is frequently presented as one of the pillars of the scientific method.

Here we are interested in the opinion revision of an agent 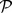 who is persuaded (or advised) by another agent 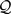 [10], [13], [52]. (Below we use the terms *opinion* and *belief* interchangeably.) We follow the known framework for representing uncertain opinions of both agents via the subjective probability theory [13]. Within this framework, the opinion of an agent about propositions (events) is described by probabilities that quantify his degree of confidence in the truth of these propositions [13]. As we argue in the next section, the standard Bayesian approach to opinion revision is inadequate for describing persuasion. Instead, here we study confirmationally-biased persuasion within the opinion combination approach developed in statistics; see [21], [30] for reviews.

We suggest a set of conditions that model cognitive aspects of confirmation bias. Essentially, those conditions formalize the intuition that the agent 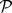 does not change his opinion if the persuasion is either far away or identical with his existing opinion [15], [60]. We then propose a simple opinion revision rule that satisfies those conditions and is consistent with the ordinary probability theory. The rule consists of two elementary operations: *averaging* the initial opinion with the persuading opinion via linear combination, and then *projecting* it onto the initial opinion. The actual existence of these two operations has an experimental support [8], [9], [18], [72,].

We demonstrate that the proposed revision rule is consistent with the *social judgment theory* [10], and reproduces the so called *change-discrepancy* relationship [10], [35], [40], [45], [69]. Furthermore, the well-studied *weighted average* approach [9], [27] for opinion revision is shown to be a particular case of our model.

Our analysis of the revision rule also reveals novel effects. In particular, it is shown that within the proposed approach, the recency effect is related to confirmation bias. Also, repeated persuasions are shown to hold certain monotonicity features, but do not obey the law of diminishing returns. We also demonstrate that the rule reproduces several basic features of the *cognitive dissonance* phenomenon and predicts new scenarios of its emergence. Finally, the so called *boomerang* (*backfire*) effect can emerge as an extreme form of confirmation bias. The effect is given a straightforward mathematical description in qualitative agreement with experiments.

The rest of this paper is organized as follows. In the next section we introduce the problem setup and provide a brief survey of relevant work, specifically focusing on inadequacy of the standard Bayesian approach to opinion revision under persuasion. In the third section we define our axioms and introduce the confirmationally biased opinion revision rule. The fourth section relates our setup to the social judgment theory. Next two sections describe how our model accounts for two basic phenomena of experimental social psychology: opinion change versus discrepancy and the order of presentation effect. The seventh section shows how our model formalizes features of cognitive dissonance, followed by analysis of opinion change under repeated persuasion. Then we study the *boomerang effect*–the agent changes his opinion not towards the persuasion, but against it– as a particular case of our approach. We summarize and conclude in the last section.

### The Set-Up and Previous Research

Consider two agents 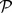 and 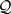. They are given an uncertain quantity (random variable) 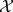 with values 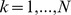, e.g. 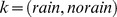, if this is a weather forecast. 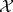 constitutes the state of the world for 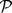 and 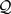. The opinions of the agents are quantified via probabilities 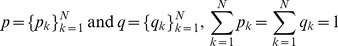
(1)for 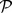 and 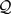 respectively.

Let us now assume that 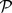 is persuaded (or advised) by 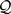. (Persuasion and advising are not completely equivalent [71]. However, in the context of our discussion it will be useful to employ both terms simultaneously stressing their commmon aspects.) Throughout this paper we assume that the state of the world does not change, and that the agents are aware of this fact. Hence, 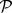 is going to change his opinion only under influence of the opinion of 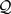, and not due to any additional knowledge about 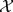 (For more details on this point see [3], [41] and the second section of File S1.)

The normative standard for opinion revision is related to the Bayesian approach. Below we discuss the main elements of the Bayesian approach, and outline certain limitations that motivates the non-Bayesian revision rule suggested in this work.

Within the Bayesian approach, the agent 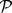 treats his own probabilistic opinion 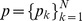 as a prior, and the probabilistic opinion 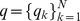 of 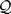 as an evidence [28], [30], [47]. Next, it is assumed that 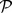 is endowed with conditional probability densities 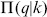, which statistically relate *q* to the world state *k*. Upon receiving the evidence from 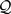, agent 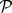 modifies his opinion from *p _k_* to 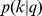 via the Bayes rule: 
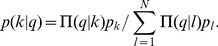
(2)


One issue with the Bayesian approach is that the assumption on the existence and availability of 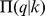 may be too strong [13], [25], [30]. Another issue is that existing empirical evidence suggests that people do not behave according to the Bayesian approach [13], [61], e.g. they demonstrate the order of presentation effect, which is generally absent within the Bayesian framework.

In the context of persuasion, the Bayesian approach (2) has two additional (and more serious) drawbacks. To explain the first drawback, let us make a generic assumption that there is a unique index 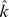 for which 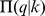 is maximized as a function of *k* (for a given *q*): 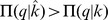 for 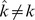.

Now consider repeated application of (2), which corresponds to the usual practice of repeated persuasion under the same opinion *q* of 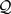. The opinion of the agent then tends to be completely polarized, i.e. 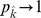 and 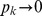 for 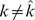. In the context of persuasion or advising, we would rather expect that under repeated persuasion the opinion of 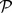 will converge to that of 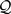.

The second issue is that, according to (2), 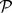 will change his opinion even if he has the same opinion as 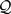: *p*  =  *q*. This feature may not be realistic: we do not expect 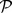 to change his opinion, if he is persuaded towards the same opinion he has already. This drawback of (2) was noted in [28]. (Ref. [28] offers a modification of the Bayesian approach that complies with this point, as shown in [28] on one particular example. However, that modification betrays the spirit of the normative Bayesianism, because it makes conditional probabilities depending on the prior probability.)

It is worthwhile to note that researchers have studied several aspects of confirmation bias by looking at certain deviations from the Bayes rule, e.g. when the conditional probability are available, but the agent does not apply the proper Bayes rule deviating from it in certain aspects [31], [51], [57], [73]. One example of this is when the (functional) form of the conditional probability is changed depending on the evidence received or on the prior probabilities. Another example is when the agent does not employ the full available evidence and selects only the evidence that can potentially confirm his prior expectations [39], [48], [67]. More generally, one has to differentiate between two aspects of the confirmation bias that can be displayed either with respect to information acquiring, or information assimilation (or both) [52]. Our study will concentrate on information assimilation aspect; first, because this aspect is not studied sufficiently well, and second, because because it seems to be more directly linked to cognitive limitations [52]. We also stress that we focus on the belief revision, and not on actions an agent might perform based on those beliefs.

### Opinion Revision Rule

We propose the following conditions that the opinion revision rule should satisfy.

**1.** The revised opinion 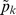 of 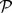 is represented as 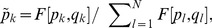
(3)where 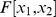 is defined over 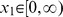 and 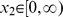. We enlarged the natural range 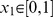 and 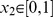, since below we plan to consider probabilities that are not necessarily normalized to 1. There are at least two reasons for doing so: First, experimental studies of opinion elicitation and revision use more general normalizations [8], [9]. For example, if the probability is elicited in percents, the overall normalization is 100. Second, and more importantly, the axioms defining subjective (or logical) probabilities leave the overall normalization as a free parameter [22].

We require that 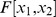 is continuous for 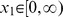 and 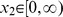 and infinitely differentiable for 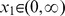 and 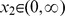. Such (or similar) conditions are needed for features that are established for certain limiting values of the arguments of *F* (cf. (5, 6)) to hold approximately whenever the arguments are close to those limiting values. *F* can also depend on model parameters, as seen below.

Eq. (3) means that 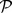 first evaluates the (non-normalized) weight 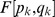 for the event *k* based *solely* on the values of *p _k_* and *q _k_*, and then applies overall normalization. A related feature of (3) is that it is local: assume that 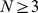 and only the probability *q*_1_ is communicated by 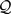 to 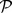. This suffices for 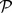 to revise his probability from *p*_1_ to 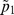, and then adjust other probabilities via renormalization:


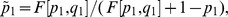


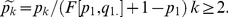
(4)


Eq. (3) can be considered as a succession of such local processes.

**2.** If 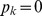 for some *k*, then 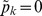: 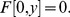
(5)


The rationale of this condition is that if 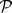 sets the probability of a certain event strictly to zero, then he sees logical (or factual) reasons for prohibiting the occurrence of this event. Hence 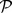 is not going to change this zero probability under persuasion.

**3.** If 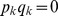 for all *k*, then 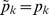: 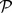 cannot be persuaded by 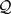 if their opinions have no overlap.

**4.** If 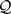
's and 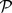
's opinions are identical, then the latter will not change his opinion: 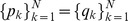 (for all *k*) leads to 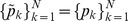. This can be written as 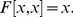
(6)


Conditions **3** and **4** are motivated by experimental results in social psychology, which state that people are not persuaded by opinions that are either very far, or very close to their initial opinion [10], [17], [69].

(Recall that we do not allow the uncertain quantity 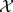 to change during the persuasion or advising. If such a change is allowed, **4** may not be natural as the following example shows. Assume that 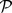 holds a probabilistic opinion 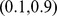 on a binary 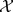. Let 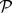 learns that 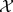 changed, but he does not know in which specific way it did. Now 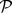 meets 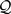 who has the same opinion 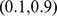. Provided that 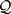 does not echo the opinion of 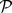, the agent 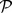 should perhaps change his opinion by decreasing the first probability (0.1) towards a smaller value, because it is likely that 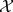 changed in that direction.)

**5.** *F* is a homogeneous function of order one: 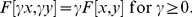
(7)


The rationale for this condition comes from the fact that (depending on the experimental situation) the subjective probability may be expressed not in normalization one (i.e. not with 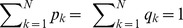), but with a different overall normalization (e.g. 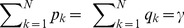) [8], [9], [22]; cf. **1**. In this light, (7) simply states that any choice of the overall normalization is consistent with the sought rule provided that it is the same for 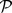 and 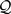. Any rescaling of the overall normalization by the factor 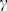 will rescale the non-normalized probability by the same factor 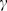; cf. (7).

**6.** Now we assume that the opinion assimilation by 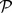 consists of two sub-processes. Both are related to heuristics of human judgement.

**6.1** 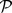 combines his opinion *linearly* with the opinion of 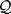 [8], [9], [18], [29], [30]: 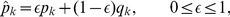
(8)where 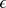 is a weight. Several mathematical interpretations of the weight 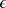 were given in statistics, where (8) emerged as one of the basic rules of probabilistic opinion combination [16], [29]; see section I of File S1. One interpretation suggested by this approach is that 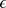 and 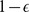 are the probabilities–from the subjective viewpoint of 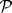 –for, respectively, *p* and *q* to be the true description of states of the world [29]: it is not known to 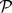 which one of these probabilities (*p* or *q*) conveys a more accurate reflection of the world state. Then 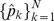 is just the marginal probability for the states of the world. There is also an alternative (normative) way of deriving (8) from maximization of an average utility that under certain natural assumptions can be shown to be the (negated) average information loss [16]; see section I of File S1 for more details.

Several qualitative factors contribute to the subjective assessment of 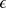. For instance, one interpretation is to relate 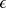 to credibility of 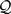 (as perceived by 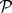): more credible 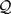 leads to a larger 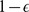 [18]. Several other factors might affect 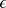: egocentric attitude of 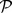 that tends to discount opinions, simply because they do not belong to him; or the fact that 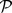 has access to internal reasons for choosing his opinion, while he is not aware of the internal reasons of 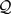 *etc* [18]. Taking into account various factors that contribute to the interpretation of 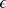, we will treat it as a free model parameter.

**6.2** Note that (8) does not satisfy conditions **2** and **3** above. We turn to the last ingredient of the sought rule, which, in particular, should achieve consistency with conditions **2** and **3**.

Toward this goal, we assume that 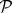 projects the linearly combined opinion 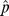 (see (8)) onto his original opinion *p*. Owing to (3), we write this transformation as 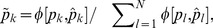
(9)where the function 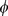 is to be determined.

The above projection operation relates to *trimming* [18], [72], a human cognitive heuristics, where 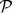 tends to neglect those aspects of 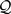
's opinion that deviate from a certain reference. In the simplest case this reference will be the existing opinion of 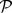.

To make the projection process (more) objective, we shall assume that it commutes with the probabilistic revision: whenever 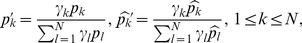
(10)where 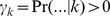 are certain conditional probabilities, 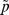 is revised via the same rule (10): 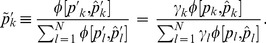
(11)


This feature means that the projection is consistent with probability theory: it does not matter whether (3) is applied before or after (10).

It is known that (9) together with (10, 11) selects a unique function [30]: 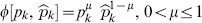
(12)where 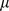 quantifies the projection strength: for 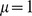 the projection is so strong that 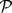 does not change his opinion at all (conservatism), while for 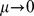, 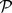 fully accepts 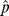 (provided that 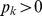 for all *k*). (The above commutativity is formally valid also for 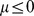 or 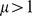, but both these cases are in conflict with (5).) In particular, 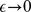 and 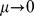 is a limiting case of a fully credulous agent that blindly follows persuasion provided that all his probabilities are non-zero. (For a sufficiently small 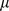, a small *p _k_* is less effective in decreasing the final probability 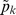; see (12). This is because 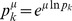 tends to zero for a fixed 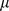 and 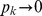, while it tends to one for a fixed *p _k_* and 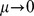. This interpaly between 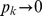 and 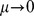 is not unnatural, since the initial opinion of a credulous agent is expected to be less relevant. The case of credulous agent is of an intrinsic interest and it does warrant further studies. However, since our main focus is confirmation bias, below we set 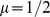 and analyze the opinion dynamics for varying 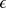.)

The final opinion revision rule reads from (12, 8, 9): 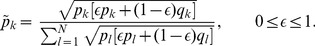
(13)


It is seen to satisfy conditions **1–5**.

(Note that the analogue of (11), 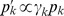, 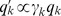 does not leave invariant the linear function (8). First averaging, 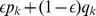 and then applying 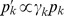, 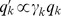 is equivalent to first applying the latter rules and then averaging with a different weight 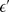. This is natural: once 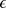 can be (in principle) interpreted as a probability it should also change under probabilistic revision process.)

The two processes were applied above in the specific order: first averaging (8), and then projection (9). We do not have any strong objective justifications for this order, although certain experiments on advising indicate on the order that led to (13) [72]. Thus, it is not excluded that the two sub-processes can be applied in the reverse order: first projection and then averaging. Then instead of (13) we get (3) with: 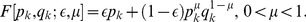
(14)


Our analysis indicates that both revision rules (13) and (14) (taken with 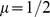) produce qualitatively similar results. Hence, we focus on (13) for the remainder of this paper.

Returning to (1), we note that *k*  =  *x* can be a continuous variable, if (for example) the forecast concerns the chance of having rain or the amount of rain. Then the respective probability densities are: 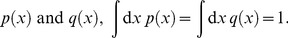
(15)


Since the revision rule (13) is continuous and differentiable (in the sense defined after (3)), it supports a smooth transition between discrete probabilities and continuous and differentiable probability densities. In particular, (13) can be written directly for densities: for 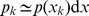 we obtain from (13) 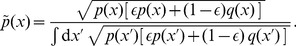
(16)


## Social Judgment Theory and Gaussian Opinions

### Opinion latitudes

Here we discuss our model in the context of the social judgment theory [59], [10], and consider several basic scenarios of opinion change under the rule (16).

According to the social judgment theory, an agent who is exposed to persuasion perceives and evaluates the presented information by comparing it with his existing attitudes (opinions). The theory further postulates that an attitude is composed of three zones, or latitudes: *acceptance*, *non-commitment* and *rejection* [10], [59]. The opinion that is most acceptable to 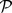, or the *anchor*, is located at the center of the latitude of acceptance. The theory states that persuasion does not change the opinion much, if the persuasive message is either very close to the anchor or falls within the latitude of rejection [10], [59]. The social judgment theory is popular, but its quantitative modeling has been rather scarce. In particular, to the best of our knowledge, there has been no attempt to develop a consistent probabilistic framework for the theory. (The literature on the social judgment theory offers some formal mathematical expressions that could be fitted to experimental data [45]. There is also a more quantitative theory [34] whose content is briefly reminded in section III of File S1.)

Let us assume that *k*  =  *x* is a continuous variable (cf. (15)) and that *p*(*x*) and *q*(*x*) are Gaussian with mean 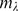 and dispersion 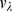 (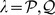): 
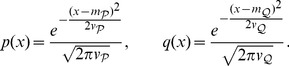
(17)


Effectively, Gaussian probabilistic opinions are produced in experiments, when the subjects are asked to generate an opinion with 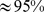 confidence in a certain interval [18]. Now we can identify the anchor with the most probable opinion 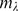, while 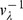 quantifies the opinion uncertainty.

The latitude of acceptance amounts to opinions not far from the anchor, while the latitude of rejection contains close-to-zero probability events, since 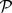 does not change his opinion on them; recall point **2** from the previous section. One can also identify the three latitudes with appropriately chosen zones in the distribution. For instance, it is plausible to define the latitudes of acceptance and rejection by, respectively, the following formulas of the 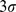 rule known in statistics 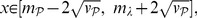
(18)

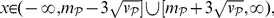
(19)where the latitude of non-commitment contains whatever is left out from (18, 19). Recall that the latitudes of acceptance, non-commitment and rejection carry (respectively) 95.4, 4.3 and 0.3% of probability.

While the definitions (18, 19) are to some extent arbitrary, they work well with the rule (16), e.g. if the opinions of 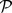 and 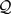 overlap only within their rejection latitudes, then neither of them can effectively change the opinion of another. Also, 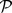 is persuaded most strongly, if the anchor of the persuasion falls into the non-commitment latitude of 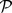. This is seen below when studying change-discrepancy relations.

### Weighted average of anchors

Next, we demonstrate that the main quantitative theory of persuasion and opinion change**–**the weighted average approach [9], [27] **–**is a particular case of our model. We assume that the opinions *p*(*x*) and *q*(*x*) are given as 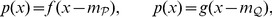
(20)

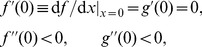
(21)where both *f*(*x*) and *g*(*x*) have a unique maximum at *x*  =  0. Hence *p*(*x*) (resp. *q*(*x*)) has a single anchor (maximally probable opinion) 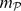 (resp. 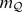); see (17) for concrete examples.

**Figure 1:**
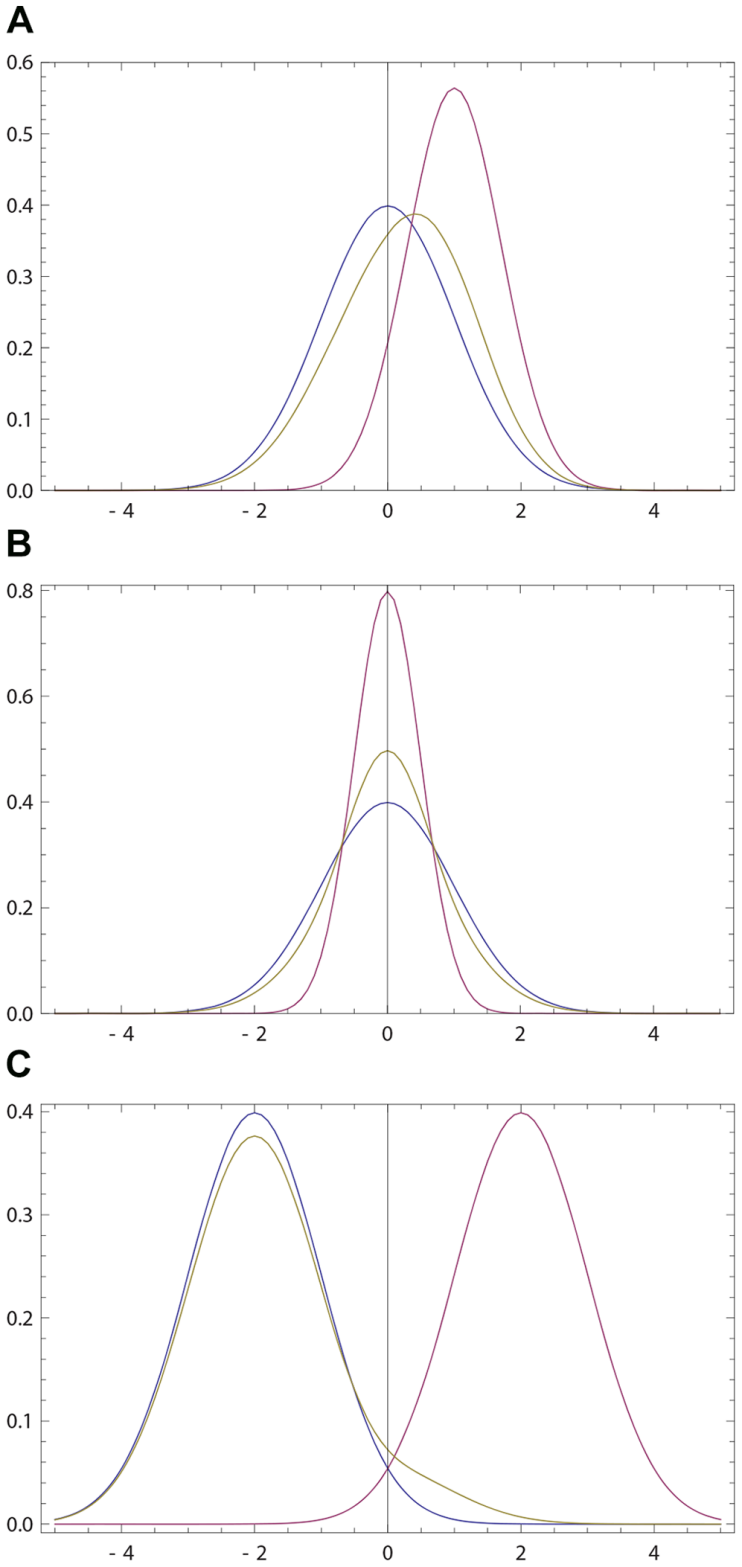
Opinions described via Gaussian densities (17). The initial opinion of 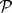 is described by Gaussian probability density *p*(*x*) (blue curve) centered at zero; see (17). The opinion of 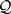 amounts to Gaussian probability density *q*(*x*) (purple curve) centered at a positive value. For all three figures continuous density *f*(*x*) (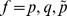) were approximated by 100 points 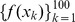, 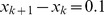. The resulting opinion 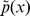 of 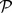 is given by (16) with 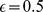 (olive curve). (a) The opinion of 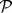 moves towards that of 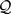; 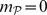, 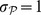, 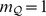, 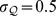. (b) The maximally probable opinion of 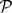 is reinforced; 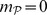, 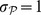, 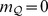, 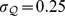. (c) The change of the opinion of 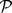 is relatively small provided that the Gaussian densities overlap only in the region of non-commitment; cf. (18), (19). Whenever the densities overlap only within the rejection range the difference between *p*(*x*) and 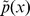 is not visible by eyes. For example, if *p*(*x*) and *q*(*x*) are Gaussian with, respectively, 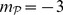, 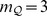, 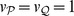, the Hellinger distance (see (30) for definition) 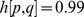 is close to maximally far, while the opinion change is small: 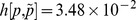.



If 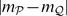 is sufficiently small, 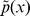 given by (20, 16) has a single anchor which is shifted towards that of *q*(*x*); see Fig. 1(a). We now look for the maximum 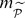 of 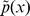 by using (20) in (16). We neglect factors of order 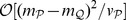 and 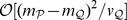 and deduce:

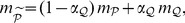
(22)

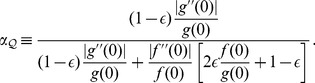
(23)


Eq. (22) is the main postulate of the weighted average approach; see [9], [27] for reviews. Here 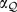 and 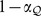 are the weights of 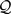 and of 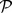, respectively. For the Gaussian case (17), we have 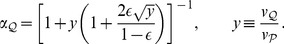
(24)


Furthermore, we have 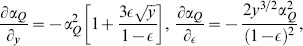
(25)


Thus, 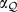
's dependence on the involved parameters is intuitively correct: it increases with the confidence 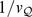 of 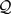, and decreases with the confidence 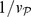 of 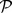. Note also that 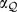 decreases with 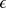.

Now let *p*(*x*) and *q*(*x*) (and hence 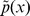) have the same maximum 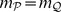, but 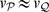; see (17). Expanding (16, 17) over 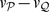 and keeping the first-order term only we get 
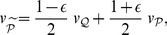
(26)where 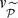 is the dispersion of (non-Gaussian) 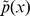. Eq. (26) implies 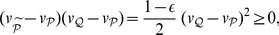
(27)i.e. if 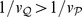 (resp. 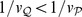), the final opinion of 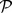 becomes more (resp. less) narrow than his initial opinion. Fig. 1(b) shows that 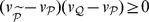 holds more generally.

Thus, the weighted average approach is a particular case of our model, where the agent 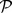 is persuaded by a slightly different opinion. Note also that our model suggests a parameter structure of the weighted average approach.

### Opinions and bump-densities

Gaussian densities (with three latitudes) do correspond to the phenomenology of social psychology. However, in certain scenarios one might need other forms of densities, e.g., when the probability is strictly zero outside of a finite support. Such opinions can be represented by bump-functions 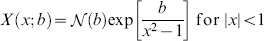
(28)

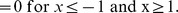
where 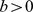 is a parameter, 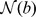 is the normalization and the support of the bump function was chosen to be 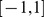 for concretness. The advantage of the bump function that is infinitely differentiable despite of having a finite support.

**Figure 2:**
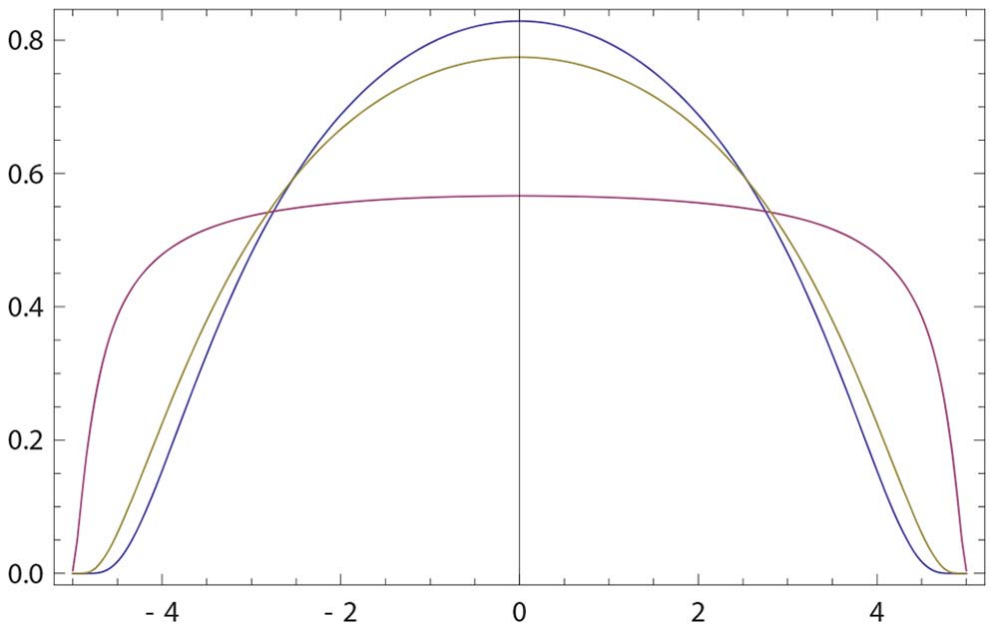
Opinions described via bump densities (28). Blue curve: the initial opinion of 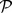 given by (28) with *b*  =  1. Purple curve: the opinion of 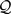 described by (28) with 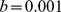. Olive curve: the resulting opinion of 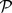 obtained via (16) with 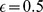.

For sufficiently large *b*, 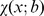 is close to a Gaussian, while for small *b*, 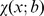 represents an opinion that is (nearly) homogeneous on the interval 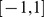; see Fig. 2. The opinion revision with bump densities follows to the general intuition of rule (16); see Fig. 2.

## Opinion Change vs Discrepancy

One of extensively studied questions in social psychology is how the opinion change is related to the discrepancy between the initial opinion and the position conveyed by the persuasive message [10], [35], [40], [45], [69]. Initial studies suggested a linear relationship between discrepancy and the opinion change [35], which agreed with the prediction of the weighted average model. Indeed, (22) yields the following linear relationship between the change in the anchor and the initial opinion discrepancy of 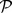 and 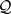: 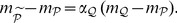
(29)


However, consequent experiments revealed that the linear regime is restricted to small discrepancies only and that the actual behavior of the opinion change as a function of the discrepancy is non-monotonic: the opinion change reaches its maximal value at some discrepancy and decreases afterward [10], [40], [45], [69].

To address this issue within our model, we need to define distance 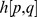 between two probability densities *p*(*x*) and *q*(*x*). Several such distances are known and standardly employed [32]. Here we select the Hellinger distance (metric) 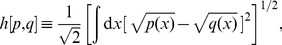
(30)

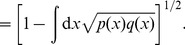
(31)


Since 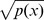 is a unit vector in the 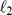 norm, Eq. (30) relates to the Euclidean (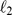-norm) distance. It is applicable to discrete probabilities by changing the integral in (30, 31) to sum. For Gaussian opinions (17) we obtain 
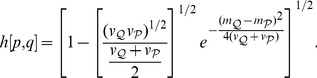
(32)


A virtue of the Hellinger distance is that it is a measure of overlap between the two densities; see (31). We stress, however, that there are other well-known distances measures in statistics [32]. All results obtained below via the Hellinger distance will be checked with one additional metric, the total variation (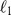-norm distance): 
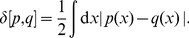
(33)


(To motivate the choice of (33), let us recall two important variational features of this distance [32]: (1) 
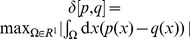
. (2) Define two (generally dependent) random variables 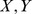 with joint probability density 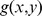 such that 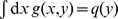, 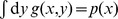. Now it holds that 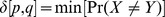, where 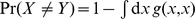, and the minimization is taken over all 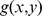 with fixed marginals equal to *p*(*x*) and *q*(*y*), respectively.)

The opinion change is characterized by the Hellinger distance 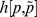 between the initial and final opinion of 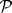, while the discrepancy is quantified by the Hellinger distance 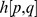 between the initial opinion of 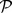 and the persuading opinion. For concreteness we assume that the opinion strengths 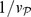 and 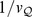 are fixed. Then 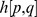 reduces to the distance 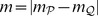 between the anchors (peaks of *p*(*x*) and *q*(*x*)); see (32).

**Figure 3:**
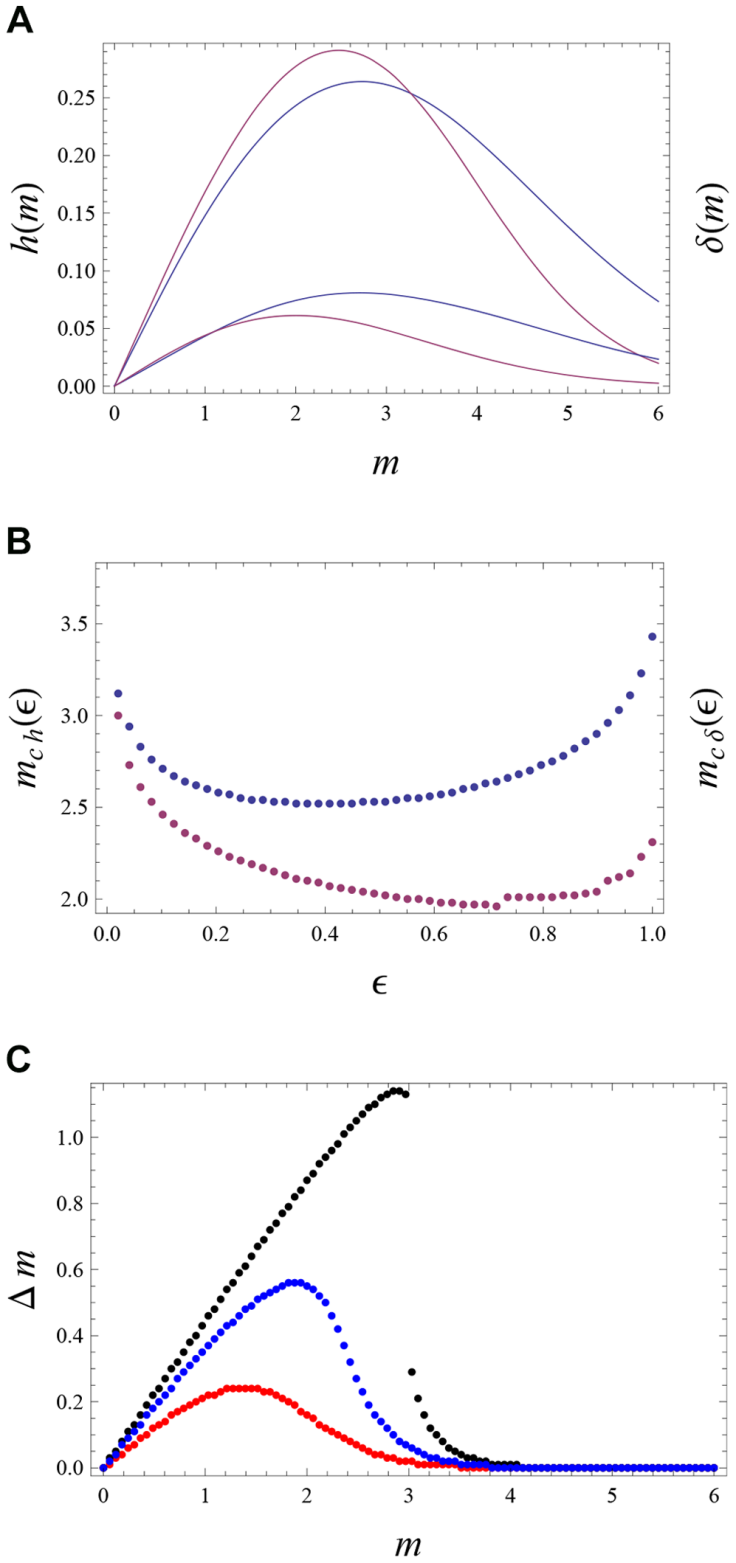
Opinion change versus discrepancy. (a) The opinion change is quantified via the Hellinger distance 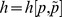 between the old and new opinion of 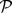 (blue curves); see (30) for the definition. For comparison we also include the total variance distance 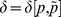 (purple curves); see (33). These two distances are plotted versus the discrepancy 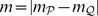. The initial opinion of the agent 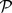 is Gaussian with 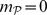 and 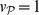; see (17). The opinion of 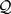 is Gaussian with 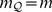 and 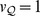. Thus *m* quantifies the initial distance between the opinions of 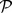 and 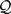. The final opinion 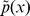 is given by (13). Different curves correspond to different 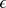. Blue curves: 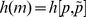 for 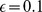 (upper curve) and 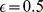 (lower curve). Purple curves: 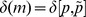 for 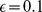 (upper curve) and 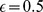 (lower curve). The maximum of *h*(*m*) (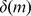) is reached at 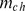 (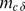). (b) 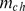 (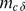) is the point where *h*(*m*) (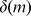) achieves its maximum as a function of *m*. Blues points: 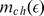 versus 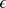 for same parameters as in (a). 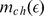 grows both for 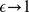 and 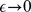, e.g. 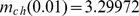, 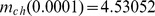, 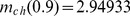, 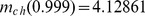. Purple points: 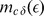 versus 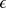 for same parameters as in (a). (c) The difference of the anchors (maximally probable values) 
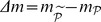
 versus 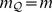 for the initial opinions of 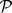 and 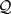 given by (17) under 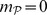, 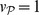, 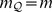 and 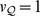. The final opinion 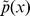 of 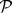 (and its maximally probable value 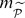) if found from (13) under 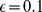 (black points), 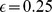 (blue points) and 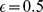 (red points).

Fig. 3(a) shows that the change 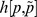 is maximal at 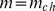; it decreases for 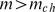, since the densities of 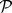 and 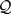 have a smaller overlap. The same behavior is shown by the total variation 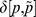 that maximizes at 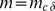; see Fig. 3(a).

The dependence of 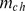 (and of 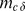) on 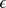 is also non-monotonic; Fig. 3(b). This is a new prediction of the model. Also, 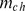 and 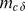 are located within the latitude of non-commitment of 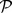 (this statement does not apply to 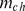, when 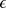 is close to 1 or 0); cf. (18, 19). This point agrees with experiments [10], [69].

Note that experiments in social psychology are typically carried out by asking the subjects to express one preferred opinion under given experimental conditions [10], [35], [40], [45], [69]. It is this single opinion that is supposed to change under persuasion. It seems reasonable to relate this single opinion to the maximally probable one (anchor) in the probabilistic set-up. Thus, in addition to calculating distances, we show in Fig. 3(c) how the final anchor 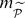 of 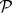 deviates from his initial anchor 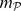.

Fig. 3(c) shows that for 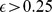, the behavior of 
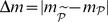
 as a function of 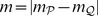 has an inverted-U shape, as expected. It is seen that 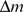 saturates to zero much faster compared to the distance 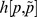. In other words, the full probability 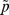 keeps changing even when the anchor does not show any change; cf. Fig. 3(c) with Fig. 3(a).

A curious phenomenon occurs for a sufficiently small 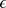; see Fig. 3(c) with 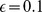. Here 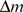 drops suddenly to a small value when *m* passes certain crticial point; Fig. 3(c). The mechanism behind this sudden change is as follows: when the main peak of *p*(*x*) shifts towards 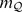, a second, sub-dominant peak of 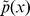 appears at a value smaller than 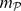. This second peak grows with *m* and at some critical value it overcomes the first peak, leading to a bistability region and an abrupt change of 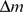. The latter arises due to a subtle interplay between the high credibility of 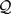 (as expressed by a relatively small value of 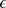) and sufficiently large discrepancy between 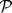 and 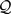 (as expressed by a relatively large value of *m*). Recall, however, that the distance 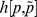 calculated via the full probability does not show any abrupt change.

The abrupt change of 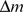 is widely discussed (and experimentally confirmed) in the attitude change literature; see [49] for a recent review. There the control variables for the attitude change**–**information and involvement [49] **–**differ from 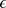 and *m*. However, one notes that the weight 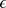 can be related to the involvement: more 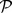 is involved into his existing attitude, larger is 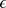, while the discrepancy *m* connects to the (new) information contained in the persuasion (*m*  =  0 naturally means zero information).

Let us finally consider a scenario where the change-discrepancy relationship is monotonic. It is realized for 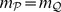 (coinciding anchors), where the distance (32) between *p*(*x*) and *q*(*x*) is controlled by 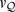 (for a fixed 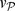). In this case, vthe change 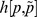 is a monotonic function of discrepancy 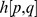: a larger discrepancy produces larger change. This example is interesting, but we are not aware of experiments that have studied the change-discrepancy relation in the case of two identical anchors.

## Order of Presentation

### Recency versus primacy

When an agent is consecutively presented with two persuasive opinions, his final opinion is sensitive to the order of presentation [10], [13], [25], [34], [35], [50], [52]. While the existence of this effect is largely established, its direction is a more convoluted matter. (Note that the order of presentation effect is not predicted by the Bayesian approach; see (2).) Some studies suggest that the first opinion matters more (primacy effect), whereas other studies advocate that the last interaction is more important (recency effect). While it is not completely clear which experimentally (un)controlled factors are responsible for primacy and recency, there is a widespread tendency of relating the primacy effect to confirmation bias [13], [52]. This relation involves a qualitative argument that we scrutinize below.

We now define the order of presentation effect in our situation. The agent 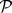 interacts first with 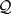 (with probability density *q*(*x*)), then with 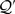 with probability density 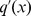. To ensure that we compare *only* the order of 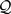 and 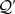 and not different magnitudes of influences coming from them, we take both interactions to have the same parameter 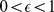. Moreover, we make 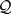 and 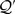 symmetric with respect to each other and with respect to 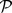, e.g. if *p*(*x*), *q*(*x*) and 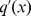 are given by (17) we assume 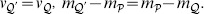
(34)


We would like to know whether the final opinion 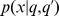 of 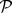 is closer to *q*(*x*) (primacy) or to 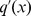 (recency).

In the present model (and for 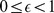), the final opinion 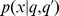 is *always* closer to the last opinion 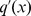, both in terms of maximally probable value and distance. In other words, the model unequivocally predicts the recency effect. In terms of the Hellinger distance (30) 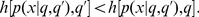
(35)


**Figure 4:**
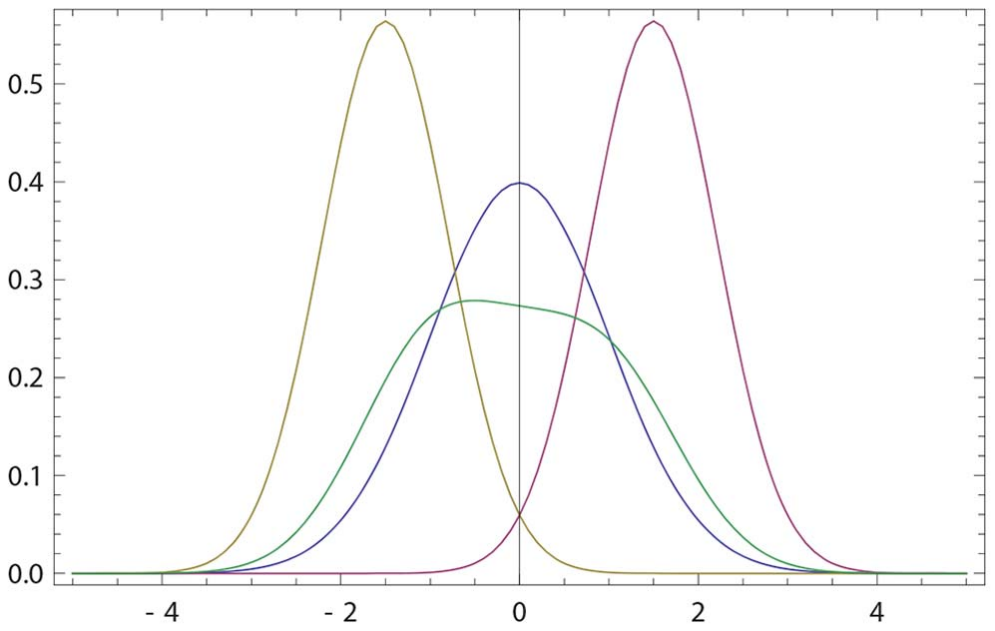
Order of presentation effect. Blue curve: The initial opinion of 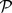 is described by Gaussian probability density *p*(*x*) with 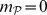 and 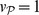; see (17). Purple (resp. olive) curve: the initial opinion of 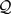 (resp. 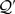) are given by (17) with 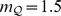 (resp. 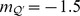) and 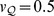 (resp. 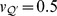). Green curve: the resulting opinion of 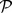 after interacting first with 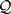 and then with 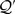. Both interactions use 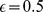. The final opinion of 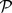 is inclined to the most recent opinion (that of 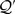) both with respect to its maximally probable value and distance. The final opinion of 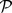 has a larger width than the initial one.

See Fig. 4 for an example (In our model primacy effect exists in the boomerang regime 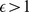; see below.)

To illustrate (35) analytically on a specific example, consider the following (binary) probabilistic opinion of 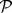, 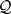 and 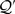


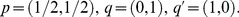
(36)



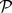
 is completely ignorant about the value of the binary variable, while 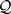 and 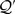 are fully convinced in their opposite beliefs. If 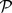 interacts first with 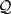 and then with 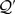 (both interactions are given by (13) with 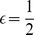), the opinion of 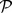 becomes 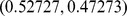. This is closer to the last opinion (that of 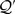).

The predicted recency effect in our model seems rather counterintuitive. Indeed, since the first interaction shifts the opinion of 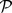 towards that of 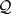, one would think that the second interaction with 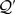 should influences 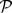
's opinion less, due to a smaller overlap between the opinions of 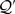 and 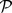 before the second interaction. In fact, this is the standard argument that relates primacy effect to the confirmation bias [13], [52]: the first interaction shapes the opinion of 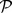 and makes him confirmationally biased against the second opinion. This argument does not apply to the present model due to the following reason: even though the first interaction shifts 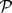
's anchor towards 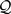
's opinion, it also deforms the shape of the opinion; see Fig. 1(a). And the deformation produced by our revision rule happens to favor the second interaction more.

To get a deeper understanding of the recency effect, let us expand (13) for small 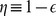:



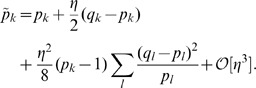
(37)


If now 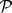 interacts with an agent 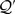 having opinion 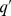, the resulting opinion 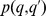 reads from (37): 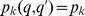


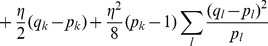


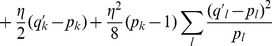


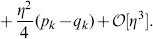
(38)


Hence in this limit 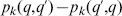 depends only on 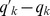 (and not e.g. on 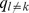): 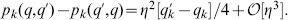
(39)


It is seen that the more probable persuasive opinion (e.g. the opinion of 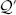 if 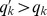) changes the opinion of 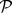 if it comes later. This implies the recency effect. Indeed, due to symmetry conditions for checking the order of presentation effect we can also look at 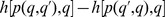. Using (39) we get for this quantity: 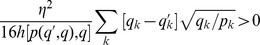, again due to symmetry conditions.

Note that this argument on recency directly extends to more general situations, where the agent is exposed to different opinions multiple times. For instance, consider an exposure sequence 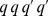 and its reverse 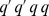. It can be shown that the model predicts a recency effect in this scenario as well. For this case, we get instead of (39): 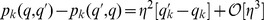.

Note that the primacy-recency effect is only one (though important!) instance of contextual and non-commutative phenomena in psychology; see [11], [66] and references therein. Hence in section IV of File S1 we study a related (though somewhat less interesting) order of presentation effect, while below we discuss our findings in the context of experimental results.

### Experimental studies of order of presentation effect

We now discuss our findings in this section in the context of experimental results on primacy and recency. The latter can be roughly divided into several group: persuasion tasks [10], [50], symbol recalling [70], inference tasks [34], and impression formation [7], [9]. In all those situations one generally observes both primacy and recency, though in different proportions and under different conditions [34]. Generally, the recency effect is observed whenever the *retention* time (the time between the last stimulus and the data taking) is short. If this time is sufficiently long, however, the recency effect changes to the primacy effect [10], [50], [62], [70]. The general interpretation of these results is that there are two different processes involved, which operate on different time-scales. These processes can be conventionally related to short-term and long-term memory [70], with the primacy effect related to the long-term memory. In our model the longer time process is absent. Hence, it is natural that we see only the recency effect. The prevalence of recency effects is also seen in inference tasks, where the analogue of the short retention time is the incremental (step-by-step) opinion revision strategy [34].

At this point, let us remind the importance of symmetry conditions [such as (34)] for observing a *genuine* order of presentation effect. Indeed, several experimental studies**–**in particular those on impression formation**–**suggest that the order of presentation exists *due* to different conditions in the first versus the second interaction [7], [10], [34], [68,]. (In our context, this means different parameters 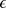 and 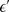 for each interaction). For instance, Refs. [7], [10] argue that the primacy effect is frequently caused by attention decrement (the first action/interaction gets more attention); see also [68] in this context. This effect is trivially described by our model, if we assume 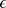 to be sufficiently smaller than 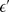. In related experiments, it was shown that if the attention devoted to two interactions is balanced, the recency effect results [33], which is consistent with the prediction of our model.

At the same time, in another interesting study based on subjective probability revision, where the authors had taken special measures for minimizing the attention decrement, the results indicated a primacy effect [55].

We close this section by underlining the advantages and drawbacks of the present model concerning the primacy-recency effect: the main advantage is that it demonstrates the recency effect and shows that the well-known argument on relating confirmation bias to primacy does not hold generally. The main drawback is that the model does not involve processes that are supposedly responsible for the experimentally observed interplay between recency and primacy. In the concluding section we discuss possible extensions of the model that can account for this interplay.

### Cognitive Dissonance

Consider an agent whose opinion probability density has two peaks on widely separated events. Such a density**–**with the most probable opinion being different from the average**–**is indicative of cognitive dissonance, where the agent believes in mutually conflicting things [10], [26].

The main qualitative scenario for the emergence of cognitive dissonance is when an agent**–**who initially holds a probabilistic opinion with a single peak**–**is exposed to a conflicting information coming from a sufficiently credible source [10], [26]. We now describe this scenario quantitatively.

**Figure 5:**
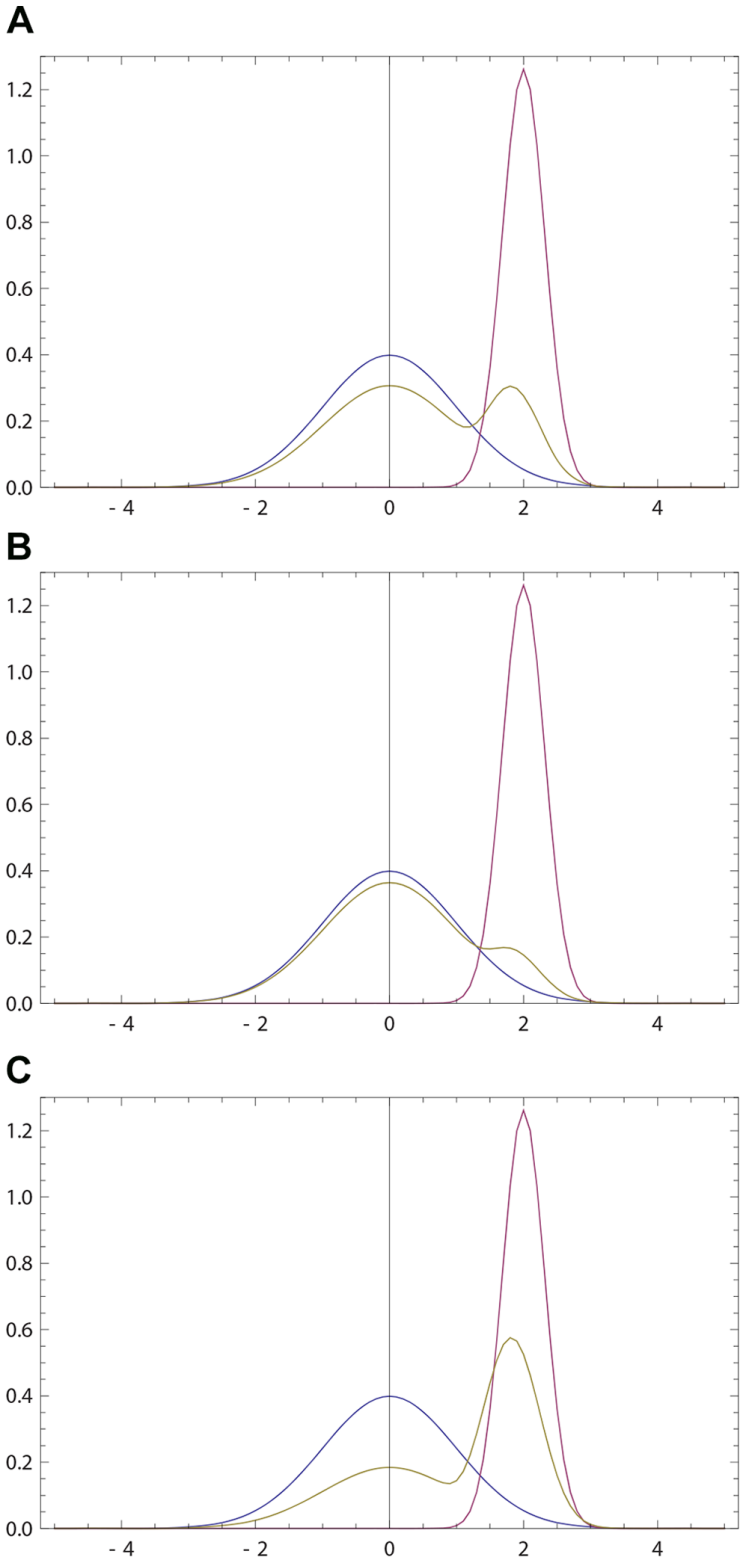
Cognitive dissonance. (a) Blue (resp. purple) curve: the initial opinion of agent 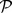 (resp. 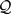) described by probability density *p*(*x*) (resp. *q*(*x*)). Olive curve: the final opinion 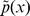 of 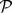 as given by (16) with 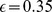. Here *p*(*x*) and *q*(*x*) are defined by (17) with 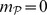, 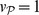, 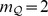, 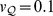. The final opinion develops two peaks of comparable height (cognitive dissonance). (b) Avoiding the cognitive dissonance due to a larger 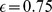: the second peak is much smaller (other parameters are those of (a)). (c) Avoiding the cognitive dissonance due to a smaller 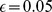: the first peak is much smaller (other parameters are those of (a)).

Consider again the opinion revision model (16, 17), and assume that 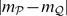 is neither very large nor very small (in both these cases no serious opinion change is expected), 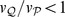 (self-assured persuasive opinion) and 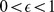. In this case, we get two peaks (anchors) for the final density 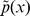. The first peak is very close to the initial anchor of *p*(*x*), while the second closer to the anchor of *q*(*x*); see Fig. 5(a). Thus, persuasion from 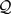 whose opinion is sufficiently narrow and is centered sufficiently close (but not too close) to 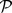
's initial anchor leads to cognitive dissonance: 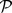 holds simultaneously two different anchors, the old one and the one induced by 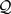.

There are 3 options for reducing cognitive dissonance:

*(i)* Increase 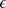 making it closer to 1, i.e. making 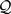 less credible; see Fig. 5(b).

*(ii)* Decrease the width of the initial opinion of 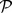.

*(iii)* Decrease 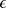 making 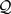 more credible. In this last case, the second peak of 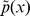 (the one close to the anchor of 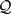) will be dominant; see Fig. 5(c).

To understand the mechanism of the cognitive dissonance as described by this model, let us start from (1) and assume for simplicity that the opinion of 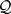 is certain: 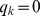 for 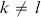 and 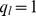. We get from (13): 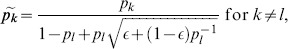
(40)

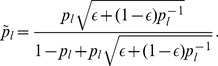
(41)


Now 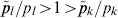, where 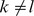; hence even if *l* was on the tail of 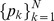, it is possible to make it a local (or even the global) maximum of 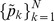 provided that 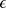 is not close to 1.

The existence of at least two widely different probable opinions is only one aspect of cognitive dissonance [10], [26]. Another aspect (sometimes called Freud-Festinger's law) is that people tend to avoid cognitive dissonance: if in their action they choose one of the two options (i.e. one of two peaks of the subjective probability), they re-write the history of their opinion revision so that the chosen option becomes the most probable one [10], [26]. This aspect of cognitive dissonance found applications in economics and decision making [2], [73]. The above points *(i)* **–** *(iii)* provide concrete scenarios for a such re-writing.

## Repeated Persuasion

Here we analyze the opinion dynamics under repeated persuasion attempts. Our motivation for studying this problem is that repeated exposure to the same opinion is generally believed to be more persuasive than a single exposure.

Under certain conditions (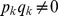, for all *k* and 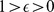) we show that the target opinion converges to the persuading opinion after sufficient number of repetition. Below we also examine how exactly this convergence takes place.

Assume that 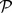 revises his opinion repeatedly with the same opinion of 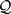. Eq. (13) implies (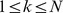) 
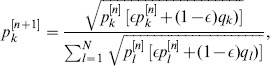
(42)where 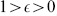, and 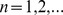 is the discrete time. For simplicity, we assume 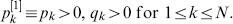
(43)


Eq. (42) admits only one fixed point 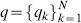. Section VI of File S1 shows that for any convex, 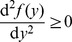, function *f*(*y*) one has 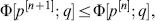
(44)

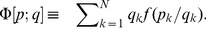
(45)


Hence 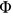 is a Lyapunov function of (42). Since 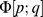 is a convex function of *p*, 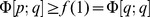 and *f*(1) is the unique global minimum of 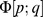. Section VI of File S1 shows that the equality sign in (45) holds ony for 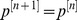. Thus 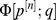 monotonically decays to 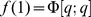 showing that the fixed point *q* is globally stable. More generally, the convergence reads: 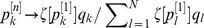, where 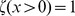 and 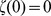.

To illustrate (44, 45), one can take 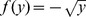. Then (44) amounts to decaying Hellinger distance (30). Many other reasonable measures of distance are obtained under various choices of *f*. For instance, 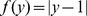 amounts to decaying total variation distance (33), while 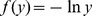 leads to the decaying relative entropy (Kullback-Leibler entropy).

As expected, 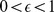 influences the convergence time. We checked that this time is an increasing function of 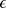, as expected. In section VI of File S1 we also show that the convergence to the fixed point respects the Le Chatelier principle known in thermodynamics [4]: the probabilities of those events that are overestimated from the viewpoint of 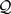 (i.e. 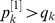) tend to decay in the discrete time. Likewise, probabilities of the underestimated events (i.e. 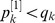) increase in time.

Let us consider the Hellinger distance 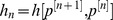 between two consecutive opinions of 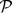 evolving as in (42). It is now possible that 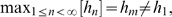
(46)i.e. the largest change of the opinion of 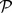 comes not from the first, but from one of intermediate persuasions. A simple example of this situation is realized for *N*  =  3, an initial probability vector 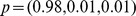 and 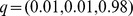 in (43). We then apply (42) under 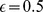. The consecutive Hellinger distances read 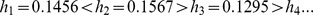. Hence the second persuasion changes the opinion more than others. For this to hold, the initial opinion *p* of 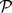 has to be far from the opinion *q* of 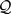. Otherwise, we get a more expected behavior 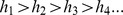 meaning that the first persuasion leads to the largest change.

(The message of (46) is confirmed by using the discrete version 
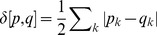
 of the distance (33). Define 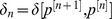. Then with 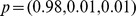 and 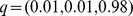 we get 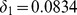, 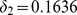, 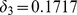, 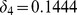.)

We conclude by stressing that while repeated persuasions drive the opinion to its fixed point monotonically in the number of repetitions, it is generally not true that the first persuasion causes the largest opinion change, i.e. the law of diminishing returns does not hold. To obtain the largest opinion change, one should carefully choose the number of repetitions.

Finally, note that the framework of (42) can be applied to studying mutual persuasion (consensus reaching). This is described in Section VII of File S1; see also [23] in this context.

## Boomerang (Backfire) Effect

### Definition of the effect

The *boomerang* or *backfire* effect refers to the empirical observation that sometimes persuasion yields the opposite effect: the persuaded agent 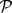 moves his opinion away from the opinion of the persuading agent, 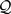, i.e. he enforces his old opinion [53], [58], [64], [69]. Early literature on social psychology proposed that the boomerang effect may be due to persuading opinions placed in the latitude of rejection [69], but this was not confirmed experimentally [40].

Experimental studies indicate that the boomerang effect is frequently related with opinion formation in an affective state, where there are emotional reasons for (not) changing the opinion. For example, a clear evidence of the boomerang effect is observed when the persuasion contains insulting language [1]. Another interesting example is when the subjects had already announced their opinion publicly, and were not only reluctant to change it (as for the usual conservatism), but even enforced it on the light of the contrary evidence [64] (in these experiments, the subjects who did not make their opinion public behaved without the boomerang effect). A similar situation is realized for voters who decided to support a certain candidate. After hearing that the candidate is criticized, the voters display a boomerang response to this criticism and thereby increase their support [53], [58].

### Opinion revision rule

We now suggest a simple modification of our model that accounts for the basic phenomenology of the boomerang effect.

Recall our discussion (around (8)) of various psychological and social factors that can contribute into the weight 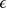. In particular, increasing the credibility of 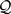 leads to a larger 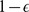. Imagine now that 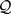 has such a low credibility that 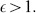
(47)


Recall that 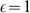 means a special point, where no change of opinion of 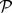 is possible whatsoever; cf. (13).

After analytical continuation of (13) for 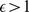, the opinion revision rule reads 
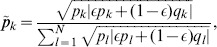
(48)


with obvious generalization to probability densities. The absolute values in (48) are necessary to ensure the positivity of probabilities.

It is possible to *derive* (rather simply postulate) (48). Toward this end, let us return to the point **6.1** and (8). During the opinion combination step, 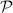 forms 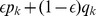 which in view of 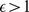 can take negative values and hence is a signed measure. Signed measures have all formal features of probability besides positivity [6], [14], [19], [65]; see section V of File S1 for details. There is no generally accepted probabilistic interpretation of signed measures, but in section V of File S1 we make a step towards such an interpretaion. There we propose to look at a signed measure as a partial expectation value defined via joint probability of the world's states and certain hidden degrees of freedom (e.g. emotional states). After plausible assumptions, the marginal probability of the world's states is deduced to be 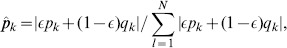
(49)


We obtain (48) after applying (9, 10) to (49).

### Scenarios of opinion change

**Figure 6:**
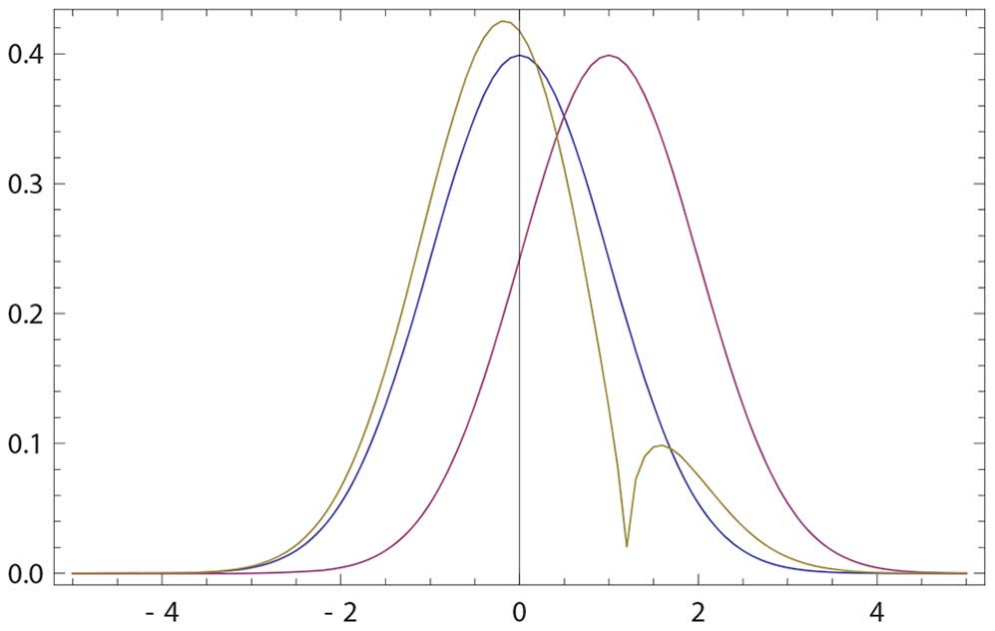
Opinion change in the boomerang regime. Blue (resp. purple) curve: the initial opinion of agent 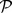 (resp. 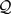) described by probability density *p*(*x*) (resp. *q*(*x*)). Olive curve: the final opinion 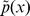 of 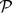 given by (16) with 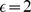. Here *p*(*x*) and *q*(*x*) are given by (17) with 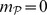 and 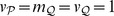. The anchor (maximally probable opinion) of 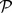 not only moves away from the anchor of 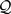; but it is also enhanced: the (biggest) peak of 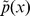 is larger than that of *p*(*x*). The second (smaller) peak of 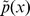 arises because the initial probability of 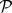 located to the right from the anchor 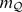 of 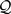, moves away from 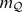; 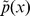 gets a local minimum close to 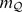.

**Figure 7:**
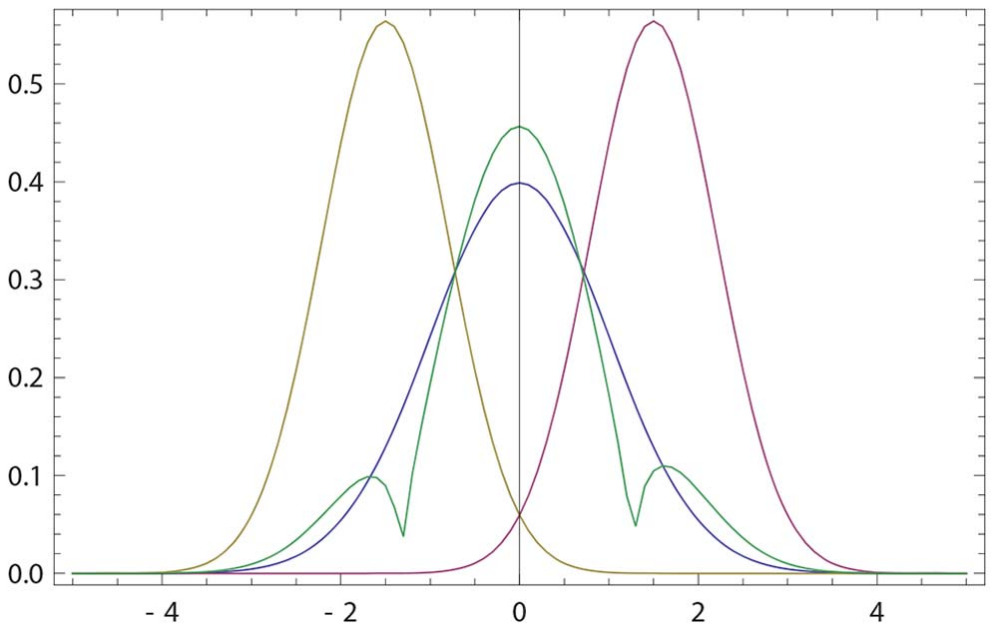
Order of presentation effect in the boomerang regime. The same as in Fig. **4** but for 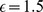 (boomerang regime). Now the final opinion of 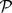 is inclined to the first opinion (that of 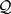) with respect to the distance. The initial maximally probable opinion of 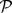 is still maximally probable. Moreover, its probability has increased and the width around it has decreased. The final opinion has 3 peaks.

According to (47, 48) those opinions of 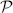 which are within the overlap between *p* and *q* (i.e 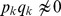) get their probability decreased if 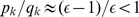, i.e. if the initial *p _k_* was already smaller than *q _k_*. In this sense, 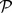 moves his opinion away from that of 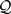. Hence for continuous densities *p*(*x*) and *q*(*x*) there will be a point *x*_0_, where 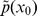 is close to 0. This point is seen in Figs. 6 and 7.

Fig. 6 illustrates the shape of 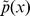 produced by (48) for initially Gaussian opinions (17) of 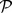 and 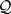. It is seen that 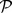
's anchor moves away from 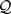
's anchor, while the width of 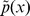 around the anchor is more narrow than that of *p*(*x*); cf. with Fig. 4. To illustrate these points analytically, we return to (29, 24, 24) that for 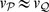 and 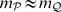 predict 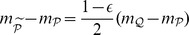: for 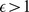, 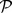
's anchor drifts away from 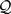
's anchor.

Likewise, whenever the two anchors are equal, 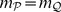, inequality (27) is reversed in the boomerang regime (47).

Let us now consider the impact of the presentation order under this settings. We saw that for 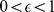 the model predicts recency effect. For 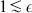 we expect the recency effect is still effective as implied by the argument (39). However, the situation changes drastically for 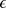 sufficiently larger than 1, as indicated in Fig. 7. Now the primacy effect dominates, i.e. instead of (35) we get the opposite inequality. Fig. 7 also shows that interaction with two contradicting opinions (in the boomerang regime) enforces the initial anchor of 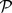.

To understand the primacy-recency effect analytically, consider the example (36), and recall that 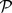 interacts first with 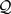 and then with 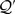 with the same parameter 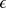. The resulting opinion 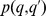 of 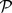 reads: 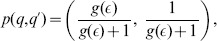
(50)

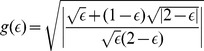
(51)


**Figure 8:**
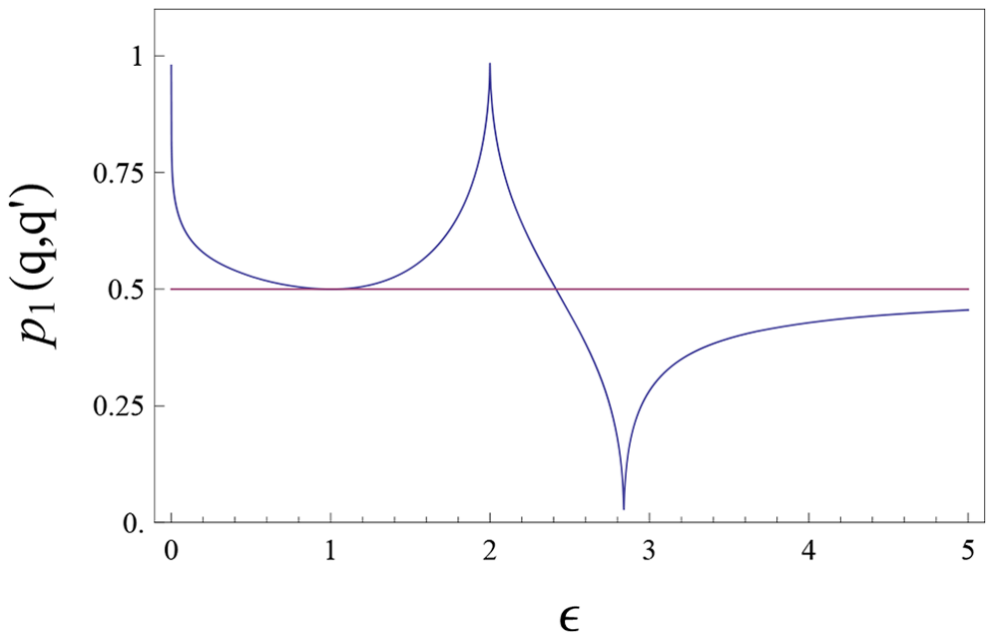
Illustration of the order of presentation effect in the boomerang regime. 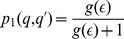
 given by (50, 51) versus 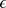.

Fig. 8 shows how 
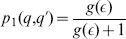
 behaves as a function of 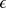. The recency effect holds for 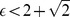; for 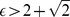 we get primacy. Similar results are obtained for initially Gaussian opinions.

Thus, in the present model, the primacy effect (relevance of the first opinion) can be related to the boomerang effect.

We now examine the emergence of cognitive dissonance in the boomerang regime 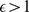. Our results indicate that in this regime the agent is more susceptible to cognitive dissonance; cf. Fig. 6 with Figs. 1. The mechanism of the increased susceptibility is explained in Fig. 6: 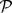
's opinion splits easier, since the probability mass moves away (in different directions) from the anchor of 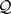.

**Figure 9:**
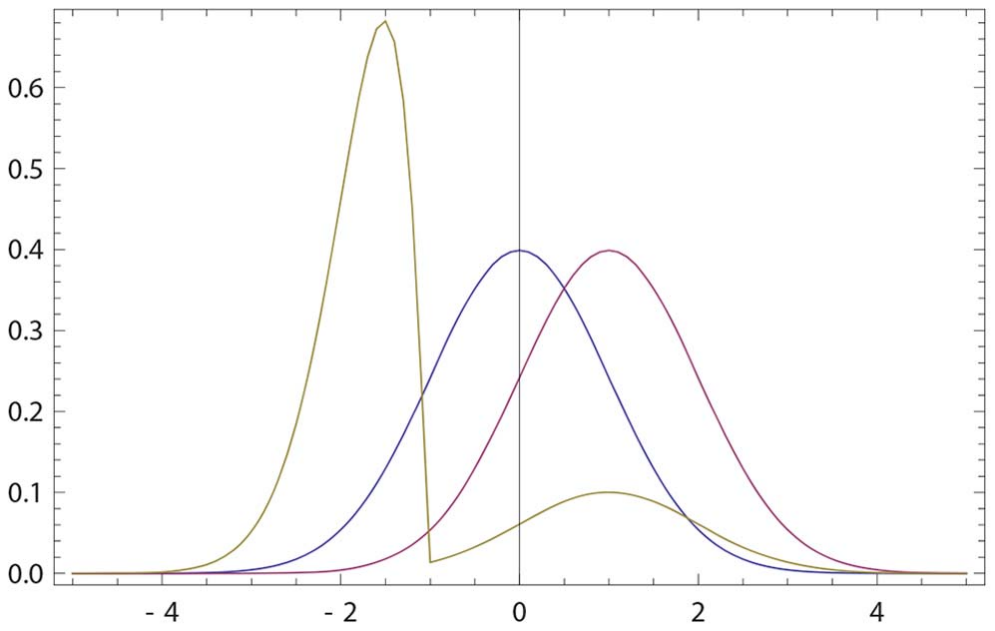
Repeated persuasion in the boomerang regime. Blue (resp. purple) curve: the initial opinion of agent 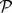 (resp. 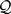) described by probability density *p*(*x*) (resp. *q*(*x*)) as given by (17) with 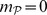, 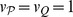, 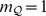. Olive curve: the opinion of 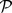 after 50 iterations (52) with 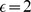.

Let us now assume that 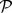 repeatedly interacts with the same opinion of 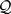 [cf. (42)]: 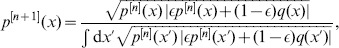
(52)where 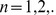 is the discrete time. Starting from initially Gaussian opinion, 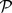 develops two well-separated peaks, which is another manifestation of cognitive dissonance: the smaller peak moves towards the anchor of 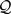 and finally places itself within the acceptance latitude of 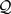, where the larger peak becomes more narrow and drifts away from *q*(*x*); see Fig. 9. After many iterations (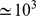 for parameters of Fig. 9) the larger peak places itself within the rejection latitude of 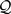, at which point 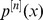 stops changing (stationary opinion). The above scenario suggests that in the boomerang regime there is a finite probability that the target agent will eventually be persuaded after repeated exposure to the same opinion.

Let us mention an experimental work that is relevant to our discussion above. Ref. [58] carried out experiments with subjects displaying boomerang effect, where each subject was exposed to sufficiently many different (but still similar) persuasive opinions. It was found that, sooner or later, the subjects exit the boomerang regime, i.e. they start to follow the persuasion [58]. Our set-up is somewhat different in that the subject (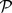) is repeatedly exposed to the same persuading opinion. Modulo this difference, our conclusion is similar to the experimental finding: the agent starts following the persuasion with a certain probability.

## Discussion

We presented a new model for opinion revision in the presence of confirmation bias. The model has three inputs: the subjective probabilistic opinions of the target agent 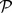 and a persuading (advising) agent 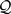, and the weight of 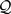 as perceived by 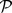.

The basic idea of the opinion revision rule is that no opinion change is expected if the persuasion is either too far or too close to the already existing opinion [15], [36], [60]. The opinion revision rule is not Bayesian, because the standard Bayesian approach does not apply to processes of persuasion and advising; see the second section for more details.

The model accounts for several key empirical observations reported in social psychology and quantitatively interpreted within the social judgment theory. In particular, the model allows to formalize the concept of opinion latitudes, explains the structure of the weighted average approach to opinion formation, and relates the initial discrepancy (between the opinions of 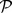 and 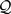) to the magnitude of the opinion change (shown by 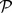). In all these cases our model extends and clarifies previous empiric results, e.g. it elucidates the difference between monotonic and non-monotonic change-discrepancy relations, identifies conditions under which the opinion change is sudden, as well as provides a deeper perspective on the weighted average approach.

New effects predicted by the model are summarized as follows.

*(i)* For the order of presentation set-up (and outside of the boomerang regime) the model displays recency effect. We suggested that the standard argument that relates confirmation bias to the primacy effect does not work in this model. In this context we recall a widespread viewpoint that *both* recency and primacy relate to (normative) irrationality; see e.g. [13]. However, the information which came later is generally more relevant for predicting future. Hence recency can be more rational than primacy.

In many experimental set-ups the recency changes to primacy upon increasing the retention time; see e.g. [70]. Our model demonstrates the primacy effect only in the boomerang regime (i.e. only in the special case). Hence, in future it needs to be extended by involving additional mechanisms, e.g. those related to “long-term memory” processes which could be responsible for the above experimental fact. Recall in this context there are several other theoretical approaches that address the primacy-recency difference [11], [34], [42], [56], [66].

*(ii)* The model can be used to describe the phenomenon of cognitive dissonance and to formalize the main scenario of its emergence.

*(iii)* Repeated persuasions display several features implying monotonous change of the target opinion towards the persuading opinion. However, the opinion changes do not obey the law of diminishing returns, or in other words, the first persuasion is not always leads to the largest change. These findings may contribute to better understanding the widespread use of repeated persuasions.

*(iv)* We proposed that the boomerang effect is related to the limit of this model, where the credibility of persuasion is (very) low. A straightforward implementation of this assumption led us to a revision rule that does describe several key observational features of the boomerang effect and predicts new ones; e.g. that in the boomerang regime the agent can be prone to primacy effect and to cognitive dissonance. There are, however, several open problems with the opinion revision rule in the boomerang regime. They should motivate future developments of this model. One problem concerns relations of the revision rule with signed measures that at a preliminary level were outlined in section V of File S1. Another problem is that the revision rule in the boomerang regime (and only there) is not completely smooth, since it includes the function 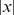, whose second derivative is singular. We do hope to clarify these points in future.

In this paper we restricted ourselves by studying few (two or three) interacting agents with opinions described via subjective probabilities. However, these probabilities can also represent an ensemble of agents each one having a fixed (single) opinion, a useful viewpoint on subjective probabilities advocated in Ref. [37]. In future we plan to explore this point and also address the opinion dynamics for collectives of agents. This last aspect was recently extensively studied via methods of statistical physics; see [20], [63] for reviews.

## Supporting Information

File S1 (PDF)
