# Supplementary material for: Opinion Dynamics with Confirmation Bias
Source: PLoS One. 2014 Jul 9;9(7):e99557. doi: 10.1371/journal.pone.0099557 (PMC4090078; doi:10.1371/journal.pone.0099557)
Supplement: File S1 — (PDF) [file pone.0099557.s001.pdf]

*Supporting Information for*

# Opinion Dynamics with Confirmation Bias

Armen E. Allahverdyan[1] and Aram Galstyan[2]

[1] *Yerevan Physics Institute,*

*Alikhanian Brothers Street 2,*

*Yerevan 375036, Armenia,*

[2] *USC Information Sciences Institute,*

*4676 Admiralty Way,*

*Marina del Rey, CA 90292, USA*

## I. LINEAR METHOD OF OPINION COMBINATION

Consider two agents with probabilistic opinions $p = \{p_k\}_{k=1}^N$ and $q = \{q_k\}_{k=1}^N$, respectively. A decision maker (which, in particular, may coincide with one of the agents) wants to combine these opinions together, hoping to get a more reliable opinion.

The linear method of combining $p$ and $q$ into the opinion $d$ of the decision maker amounts to [1, 2]:

$$d_k = w_{\mathcal{P}} p_k + w_{\mathcal{Q}} q_k, \quad w_{\mathcal{P}} + w_{\mathcal{Q}} = 1, \quad 1 \leq k \leq N, \tag{1}$$

where $w_{\mathcal{P}}$ and $w_{\mathcal{Q}}$ are positive weights that quantify the importance of each agent for the decision maker.

There are several different axioms on the decision making process that can lead to (1). We present here the main aspects of them; see [1] for more details.

Eq. (1) is a consequence of the marginalization requirement, where the opinion combination is required to commute with marginalization of probabilities [1]. This feature is relevant if the decision maker is interested only in one part of the agent's full opinion. Alternatively, (1) emerges from demanding that $d_k$ be a function of $p_k$ and $q_k$ only: $d_k = F(p_k, q_k)$ [1].

An alternative (and perhaps more elegant) way of deriving (1) is to proceed via a variational principle [3]. Let us slightly generalize (and specify) the situation assuming that we are given $n$ probabilities $p^{[\alpha]} = \{p_k^{[\alpha]}\}_{k=1}^N$ ($\alpha = 1, ..., n$) and corresponding weights $w^{[\alpha]}$. Each weight $w^{[\alpha]}$ denotes the probability that the corresponding $p^{[\alpha]}$ is true. Now the combined probability $d^* = \{d_k^*\}_{k=1}^N$ will be determined from the maximization of the average utility

$$\mathcal{U} = \sum_{\alpha=1}^{n} w^{[\alpha]} \sum_{k=1}^{N} u_k^{[\alpha]}(d^*)\, p_k^{[\alpha]}, \tag{2}$$

where $u_k^{[\alpha]}(d^*)$ is the partial utility. We now impose two natural conditions: $u_k^{[\alpha]}(d^*)$ is homogeneous (i.e. does not depend on $\alpha$) and local:

$$u_k^{[\alpha]}(d^*) = u(d_k^*). \tag{3}$$

There is an additional condition one should impose on $u(.)$: if there is only one component $\alpha$ (say $\alpha = 1$), then the utility $\sum_{k=1}^N u(d_k^*)\, p_k^{[1]}$ is maximized for $d = p^{[1]}$:

$$\max_{d^*} \left[ \sum_{k=1}^{N} u(d_k^*)\, p_k^{[1]} \right] = \sum_{k=1}^{N} u(p_k^{[1]})\, p_k^{[1]}. \tag{4}$$

This condition is necessary for the utility maximization to have its proper meaning.

The maximization in (4) is easily carried out with help of Lagrange multipliers producing:

$$u(d_k^*) = A\ \ln(d_k^*) \qquad A > 0, \tag{5}$$

where the positive constant $A$ can be set to one without loss of generality: $A = 1$. Using (5) in (2) and adding there a term that does not depend on $d^*$ we get for the average utility:

$$-\mathcal{U} = \sum_{\alpha=1}^{n} w^{[\alpha]} \sum_{k=1}^{N} p_k^{[\alpha]}\ \ln \frac{p_k^{[\alpha]}}{d_k^*}, \tag{6}$$

which is the averaged (over $w^{[\alpha]}$) relative entropy (Kullback-Leibler information) [3].

Now maximizing $\mathcal{U}$ over $d_k^*$ we are back to (1) [3]: $d_k^* = d_k = \sum_{\alpha=1}^{n} w^{[\alpha]} p_k^{[\alpha]}$.

## II. OPINION REVISING VERSUS UPDATING

As we stated in the main text, our set-up assumes that the state of the world is unchanged. We mention this condition explicitly so as to avoid confusion between *belief revision* (which is the focus of our work), and *belief update*, which carries a different meaning in the literature [4, 5]. Belief revision refers to the scenario where one encounters new information about the unchanged world. Belief update, on the other hand, refers to the scenario where one's belief is modified in response to changes in the state of the world [4, 5].

Here is a simple example demonstrating this difference. Consider two systems $\alpha$ and $\beta$, and assume that each of these systems can be in one of two states 0 or 1, so that the joint state (of the world) can be $(\alpha 0, \beta 0)$, $(\alpha 1, \beta 1)$, $(\alpha 1, \beta 0)$ and $(\alpha 0, \beta 1)$.

Assume that our initial belief is that one of the systems is in the state 0. Next, the state of the world changes somehow, and we receive information that $\alpha$ is in the state 1. This is going to be our *updated* opinion. It does not carry any information about $\beta$. Now, assume that with the same initial opinion we learn that the state of the world has not change, but we receive an information that $\alpha$ is in 1. Since the state of world has not change, the *revised* opinion will be that $\alpha$ is in 1 *and* $\beta$ is in 0.

## III. THE BELIEF-ADJUSTMENT MODEL

This model was proposed by Hogarth and Einhorn in [6]. It assumes discrete-time ($k = 1, 2, 3...$) dynamics for the anchor point $S_k$ as a function of the (new) evidence $x_k$ received

at step $k$ [6]:

$$S_k = S_{k-1} + w[s(x_k) - R], \tag{7}$$

where $s(.)$ refers to the evaluation of the evidence, $w > 0$ refers to the interaction strength and $R$ is a threshold parameter. The model does not operate with probabilities (though the usage of the anchor point can be traced back to the most probable opinion). Hence it also lacks a natural representation of acceptance and rejection latitudes and their interaction with the anchor. In several aspects the belief-adjustment model is similar to the weighted average approach with a special form of the weights.

Hogarth and Einhorn differentiate between Step-by-Step (SbS) and End-of-Sequence (EoS) revision strategy [6]. They also have simple/complex task, and we believe our model for opinion revision relates to the former. They also differentiate between estimation and evaluation tasks. Under SbS, their evaluation model always predicts recency in agreement with ours.

Furthermore, their model predicts primacy effect under EoS, with additional assumptions that initially the agent has not committed to any opinion. In this case, the claim is that the first piece of evidence serves as an anchor. This point corresponds to the more or less standard intuition on the relation between confirmation bias and primacy that we criticized in the main text.

## IV. ORDER OF PRESENTATION: ANOTHER ASPECT

The order of presentation effect that we discussed in the main text has another aspect (we freely use notations of the main text): assume that $\mathcal{Q}$ and $\mathcal{Q}'$ persuade $\mathcal{P}$ in the same direction—i.e. $m_{\mathcal{Q}} > m_{\mathcal{P}}$ and $m_{\mathcal{Q}'} > m_{\mathcal{P}}$—but their distances from the anchor of $\mathcal{P}$ are different: $m_{\mathcal{Q}'} > m_{\mathcal{Q}} > m_{\mathcal{P}}$. In which order should $\mathcal{Q}$ and $\mathcal{Q}'$ act to yield the maximal change in $\mathcal{P}$'s opinion? It is assumed, as in the main text, that both interactions have the same $\epsilon$ and that $v_{\mathcal{Q}'} = v_{\mathcal{Q}}$ to make the comparison unambiguous. The answer is again unique (but this time also intuitive) within the present model: the maximal change—as measured e.g. by the Hellinger distance—is achieved when the closer opinion acts first:

$$h[p(x|q,q'),p] > h[p(x|q',q),p]. \tag{8}$$

The same conclusion holds for $v_{\mathcal{Q}'} < v_{\mathcal{Q}}$ and $m_{\mathcal{Q}'} = m_{\mathcal{Q}}$, where opinion of $\mathcal{Q}'$ is more distant from the initial opinion of $\mathcal{P}$.

The message of (8) is intuitive, since the interaction of $\mathcal{P}$ with $\mathcal{Q}'$ is weaker: the interaction with $\mathcal{Q}$ prepares the ground for the subsequent action of $\mathcal{Q}'$. But there are experimental results that seemingly contradict this result [7]. They show that when the most distant message acts before the less distant one, the opinion changes more than for the reverse order. We believe that in those experiments the above condition on the same value of $\epsilon$ did not hold. This agrees with the viewpoint expressed by the authors of [7]. If $\epsilon$ and $\epsilon'$ are different, (8) does not hold anymore, and our model can account for the main result of [7].

## V. SIGNED MEASURES AND LOCAL VALUES.

Given a set of elements $\{1, ..., N\}$, a signed measure $\nu$ is an additive function on all subsets of $\{1, ..., N\}$, e.g. $\nu_{\{1,2\}} = \nu_1 + \nu_2$ and $\nu_{\{1,...,N\}} = \sum_{k=1}^{N} \nu_k$. However, in constrast to probability, signed measures need not be positive, i.e. $\nu_k < 0$ for some $k$'s.

There have been several attempts in literature to interpret signed measures as negative probabilities [8–11]. Below we provide an alternative interpretation of a signed measure $\{\nu_k\}_{k=1}^{N}$.

Thus, we try to give a tentative answer to the following question: what does it mean that the opinion of an agent is described by a signed measure $\nu$?

Let us divide

$$\{1, ..., N\} = \Omega^+ \cup \Omega^-, \tag{9}$$

so that $\nu_k \geq 0$ for $k \in \Omega^+$ and $\nu_k < 0$ for $k \in \Omega^-$. Let us assume that the state of the agent has a hidden variable that assumes two values $A > 0$ and $-A < 0$.

Given that the agent is in the positive (resp. negative) hidden state, his subjective probability for the states of the world is $\{\pi(k|A)\}_{k=1}^{N}$ (resp. $\{\pi(k| - A)\}_{k=1}^{N}$), while the probabilities to be in those hidden states are $\pi(A)$ and $\pi(-A) = 1 - \pi(A)$, respectively.

Now the signed measure $\nu$ is postulated to be the local average of the hidden state:

$$\nu_k = A\pi(k|A)\pi(A) - A\pi(k| - A)\pi(-A). \tag{10}$$

To determine $A$, $\pi(k| \pm A)$ and $\pi(\pm A)$ from within $\nu$ we introduce a simplifying assumption

(cf. (9)):

$$\pi(k|A) = 0 \quad \text{for} \quad k \in \Omega^-, \tag{11}$$

$$\pi(k|-A) = 0 \quad \text{for} \quad k \in \Omega^+, \tag{12}$$

i.e. there is a rigid correlation between the hidden state and $\Omega^{\pm}$. We now deduce from (9, 10, 11, 12):

$$A = \sum_{k\in\Omega^+} \nu_k + \sum_{k\in\Omega^-} |\nu_k|, \tag{13}$$

$$\pi(A) = \frac{1}{A}\sum_{k\in\Omega^+} \nu_k, \quad \pi(-A) = \frac{1}{A}\sum_{k\in\Omega^-} |\,\nu_k|, \tag{14}$$

$$\pi(k|A) = \frac{\nu_k}{\sum_{k\in\Omega^+}\nu_k} \quad \text{for} \quad k \in \Omega^+, \tag{15}$$

$$\pi(k|-A) = \frac{|\nu_k|}{\sum_{k\in\Omega^-}|\nu_k|} \quad \text{for} \quad k \in \Omega^-. \tag{16}$$

Once we determined the joint probability of the states of the world and the hidden state, we can easily find the marginal probability of the world's states:

$$\begin{aligned} \widehat{\nu}_k &= \pi(k|A)\pi(A) + \pi(k|-A)\pi(-A) \\ &= \frac{|\nu_k|}{\sum_{k=1}^N |\nu_k|}. \end{aligned} \tag{17}$$

Let us now relate the presented results to the description of the boomerang effect proposed in the main text. During the first (integration) step the agent $\mathcal{P}$ forms the signed measure $\epsilon p_k + (1-\epsilon)q_k$ with $\epsilon > 1$, which—according to the above analysis—is interpreted as a local value. Then one deduces as in (17) the marginal probability

$$\widehat{p}_k = |\epsilon p_k + (1-\epsilon)q_k| \Bigg/ \sum_{l=1}^N |\epsilon p_k + (1-\epsilon)q_k|\,, \tag{18}$$

which describes the subjective opinion of the agent $\mathcal{P}$ on the states of the world after the first step; cf. (49) of the main text.

## VI. LYAPUNOV FUNCTIONS FOR REPEATED PERSUASIONS

### A. Derivation

Here we show that in

$$\widetilde{p}_k = \frac{\sqrt{p_k[\epsilon p_k + (1-\epsilon)q_k]}}{\sum_{l=1}^N \sqrt{p_l[\epsilon p_l + (1-\epsilon)q_l]}}, \qquad 0 \le \epsilon \le 1, \tag{19}$$

the revised opinion $\widetilde{p} = (p_1, ..., p_N)$ is closer to $q = (q_1, ..., q_N)$.

Let us for simplicity assume that

$$p_k > 0, \quad q_k > 0 \ \text{ for } \ 1 \leq k \leq N, \tag{20}$$

and define

$$z_k \equiv p_k/q_k, \qquad \widetilde{z}_k \equiv \widetilde{p}_k/q_k. \tag{21}$$

We choose the indices $k$ such that the following ordering relations hold

$$z_1 \geq ... \geq z_N. \tag{22}$$

Eq. (19) implies

$$\widetilde{z}_k = \frac{\psi[z_k]}{\sum_{l=1}^N q_l \psi[z_l]}, \qquad k = 1, ..., N, \tag{23}$$

$$\psi[z] \equiv \sqrt{z^2\epsilon + z(1-\epsilon)}. \tag{24}$$

For $z > 0$ and $0 < \epsilon < 1$ we note the following features of $\psi[z]$:

$$\frac{\mathrm{d}\psi[z]}{\mathrm{d}z} > 0, \qquad \frac{\mathrm{d}}{\mathrm{d}z}\left(\psi[z]/z\right) < 0. \tag{25}$$

These relations imply from (22):

$$\widetilde{z}_1 \geq ... \geq \widetilde{z}_N, \tag{26}$$

$$\frac{\widetilde{z}_1}{z_1} \leq ... \leq \frac{\widetilde{z}_N}{z_N}. \tag{27}$$

Due to

$$\sum_{k=1}^N q_k \widetilde{z}_k = \sum_{k=1}^N q_k z_k = 1, \tag{28}$$

we have from (26):

$$\frac{\widetilde{z}_1}{z_1} \leq 1, \quad \frac{\widetilde{z}_N}{z_N} \geq 1. \tag{29}$$

Hence there exist such a $\theta$ $(1 \leq \theta < N)$ that

$$\frac{\widetilde{z}_1}{z_1} \leq 1, \; ...., \; \frac{\widetilde{z}_\theta}{z_\theta} \leq 1, \; \frac{\widetilde{z}_{\theta+1}}{z_{\theta+1}} \geq 1, \; ... \; \frac{\widetilde{z}_N}{z_N} \geq 1. \tag{30}$$

Eqs. (30, 28) lead to

$$\sum_{k=1}^{m} \widetilde{p}_k \le \sum_{k=1}^{m} p_k, \quad m = 1, ..., N-1. \tag{31}$$

Eqs. (22, 26, 28, 31) imply that for any convex $[f''(y) \ge 0]$ function $f(y)$ one gets [12, 13]:

$$\sum_{k=1}^{N} q_k f(\widetilde{p}/q_k) \le \sum_{k=1}^{N} q_k f(p_k/q_k). \tag{32}$$

Let us demonstrate the implication explicitly, since it is a useful exercise on features of convex functions. We define

$$Q_k \equiv \frac{f(z_k) - f(\widetilde{z}_k)}{z_k - \widetilde{z}_k}, \tag{33}$$

$$\alpha_k \equiv \sum_{l=1}^{k} p_l, \quad \widetilde{\alpha}_k \equiv \sum_{l=1}^{k} \widetilde{p}_l, \tag{34}$$

$$\alpha_0 \equiv \widetilde{\alpha}_0 \equiv 0. \tag{35}$$

Note that whenever $z_k = \widetilde{z}_k$ (for a certain $k$), we define $Q_k = f'(z_k)$ instead of (33).

We deduce from (22, 26) and from convexity of $f(y)$:

$$Q_1 \ge Q_2 \ge ... \ge Q_N. \tag{36}$$

The sought implication amounts to summation by parts:

$$\begin{aligned} &\sum_{k=1}^{N} q_k [f(z_k) - f(\widetilde{z}_k)] \\ &= \sum_{k=1}^{N} [p_k - \widetilde{p}_k] \frac{f(z_k) - f(\widetilde{z}_k)}{z_k - \widetilde{z}_k} \\ &= \sum_{k=1}^{N} Q_k [\alpha_k - \alpha_{k-1} - (\widetilde{\alpha}_k - \widetilde{\alpha}_{k-1})] \\ &= \sum_{k=1}^{N} (Q_k - Q_{k+1})(\alpha_k - \widetilde{\alpha}_k) \ge 0. \end{aligned} \tag{37}$$

The last expression is non-negative due to (33) and (31). The boundary terms in the summation by parts disappear due to $\alpha_N = \widetilde{\alpha}_N = 1$ and to (35).

Now recall that inequalities in (25) are strict. Hence if the initial conditions are chosen such that all inequalities in (22) are strict, and also if $f(y)$ is strictly convex, $f''(y) > 0$, all the inequalities leading to (37) can be made strict in the sense that whenever (37) nullifies, we conclude that $p = \widetilde{p}$.

### B. Interpretations

Eq. (26) means that if the ordering (22) stays intact. It can be given following meaning: $p_k \neq q_k$ means a disagreement between the opinions of $\mathcal{P}$ and $\mathcal{Q}$ on the probability of the event $k$. There can be two types of disagreement: overestimation ($p_k > q_k$) and underestimation ($p_k < q_k$).

Now (22) implies that there exist some $\zeta$, $1 \leq \zeta < N$, such that

$$z_1 \geq 1, \, ..., \, z_\zeta \; \geq 1, \, z_{\zeta+1} \; \leq 1, \, ..., \, z_N \; \leq \; 1. \tag{38}$$

All the events $1, ..., \zeta$ ($\zeta + 1, ..., N$) are overestimated (underestimated) from the viewpoint of $\mathcal{Q}$.

According to (38) the first event was overestimated. Its probability $p_1$ decays, as (30) shows. Likewise, the last event was underestimated and its probability $p_N$ increases; see (30). Since generally $\zeta \neq \theta$, the correlation between decay and overestimation (resp. increase and underestimation) need hold for all other events (i.e. for $1 < k < N$), but still this correlation holds in a more limited sense. Eq. (31) means that the sum of probabilities of the most overestimated event $p_1$ and its neighbours ($p_2, p_3$...) decays in time, although (say) $p_2$ may still indicate on overestimated event, but increase in time for some finite number of time-steps.

Following the classification of stability notions proposed in [13] for probability dynamics, (30) can be called the strong Le Chatelier principle. The general heuristics of this principle in thermodynamics is that [13]: *An external influence disturbing an equilibrium state of a system induces processes tending to diminish the results of the disturbance.* For the present opinion dynamics, the equilibrium state refers to $q$, while the perturbation over it can be taken to be $p$. If the disagreement is taken to be the cause of this perturbation, then the decay of the probability of the overestimated event (resp. increase for the underestimated event) makes sense from the viewpoint of the principle.

## VII. CONSENSUS REACHING.

Consider the following scenario of mutual influence where $\mathcal{P}$ is persuaded by $\mathcal{Q}$ and simultaneously $\mathcal{Q}$ is persuaded by $\mathcal{P}$. For repeated interactions, we obtain [normalization

factors are omitted]

$$p^{[n+1]}(x) \propto \sqrt{p^{[n]}(x)\,[\epsilon_{\mathcal{P}} p^{[n]}(x) + (1-\epsilon_{\mathcal{P}}) q^{[n]}(x)]}, \tag{39}$$

$$q^{[n+1]}(x) \propto \sqrt{q^{[n]}(x)\,[\epsilon_{\mathcal{Q}} q^{[n]}(x) + (1-\epsilon_{\mathcal{Q}}) p^{[n]}(x)]}. \tag{40}$$

where $\epsilon_{\mathcal{P}}$ and $\epsilon_{\mathcal{Q}}$ are the confirmation bias parameters for $\mathcal{P}$ and $\mathcal{Q}$, respectively. Ref. [14] recently studied a similar problem within the weighted average approach; see (22) of the main text.

For $n \to \infty$ recursions (39, 40) converge to a stationary density $p^{[\infty]}(x) = q^{[\infty]}(x) \equiv r(x)$, which depends on the initial states $p^{[1]}(x) = p(x)$, $q^{[1]}(x) = q(x)$, and on $\epsilon_{\mathcal{P}}$ and $\epsilon_{\mathcal{Q}}$. Let us discuss the main scenarios for the behavior of $r(x)$ assuming the Gaussian situation for initial densities:

$$p(x) = \frac{e^{-\frac{(x-m_{\mathcal{P}})^2}{2v_{\mathcal{P}}}}}{\sqrt{2\pi v_{\mathcal{P}}}}, \qquad q(x) = \frac{e^{-\frac{(x-m_{\mathcal{Q}})^2}{2v_{\mathcal{Q}}}}}{\sqrt{2\pi v_{\mathcal{Q}}}}. \tag{41}$$

Recall that $1/v_\lambda$ in (41) may be related to the amount of strength (self-confidence) present in the opinion.

**1.** This is a general feature of $r(x)$ (for the sake of concreteness we take $m_{\mathcal{P}} > m_{\mathcal{Q}}$): $r(x)$ is spread over the interval

$$x \in [m_{\mathcal{Q}} - 2\sqrt{v_{\mathcal{Q}}}, m_{\mathcal{P}} + 2\sqrt{v_{\mathcal{P}}}], \tag{42}$$

which includes the acceptance latitudes of $p(x)$ and $q(x)$; see (18,19) of the main text. Hence consensus reaching implies joining of acceptance latitudes for the agents.

**2.** *Equally biased, equally self-confident agents.* In this case $\epsilon_{\mathcal{P}} = \epsilon_{\mathcal{Q}}$ and $v_{\mathcal{P}} = v_{\mathcal{Q}}$. If $|m_{\mathcal{P}} - m_{\mathcal{Q}}|$ is not large (initial opinions are not far from each other), $r(x)$ is centered at $(m_{\mathcal{P}} + m_{\mathcal{Q}})/2$, i.e. in between of two opinions. If the initial opinions are sufficiently far from each other, $\mathcal{P}$ and $\mathcal{Q}$ do develop double-peak structure (cognitive dissonance) in their consensus opinion $r(x)$. Two peaks of $r(x)$ are located very close to $x = m_{\mathcal{P}}$ and $x = m_{\mathcal{Q}}$, respectively, meaning that each agent has now two equal maximally probable opinion (anchors): his initial opinion and the initial of the other agent. Thus if two biased agents with widely different opinions are forced to reach consensus, they are going to develop cognitive dissonance. This dissonance is decreased (or sometimes eliminated), if the agents are made less biased.

**3.** *Non-equally biased, equally self-confident agents*: $\epsilon_{\mathcal{P}} > \epsilon_{\mathcal{Q}}$ (for concreteness), but still $v_{\mathcal{P}} = v_{\mathcal{Q}}$. In the previous situation of equally biased agents, the peak of $r(x)$ (if it was unique) was located at the average opinion $(m_{\mathcal{P}} + m_{\mathcal{Q}})/2$. Now the peak of $r(x)$ is shifted towards more confirmationally biased agent $\mathcal{P}$.

The convergence of $p^{[n]}(x)$ and $q^{[n]}(x)$ towards $r(x)$ takes place in two steps: first $p^{[n]}(x)$ quickly spreads over the interval (42) without changing much its maximally probable value. After that $p^{[n]}(x) \approx r(x)$ does not change anymore, but $q^{[n]}(x)$ is gradually (i.e over a longer time) forced to reach the same maximally probable value as $p^{[n]}(x)$. Thus $\mathcal{P}$ first accepts to an extent the opinions of $\mathcal{Q}$ (as well as all intermediate opinions), but then gradually forces $\mathcal{Q}$ towards accepting his maximally probable opinion.

**4.** *Equally biased, non-equally self-confident agents*: $\epsilon_{\mathcal{P}} = \epsilon_{\mathcal{Q}}$, but $v_{\mathcal{P}} < v_{\mathcal{Q}}$. Now $\mathcal{P}$ is more self-confident. Hence in the consensus reaching, $\mathcal{P}$ forces $\mathcal{Q}$ to accept his maximally probable opinion (anchor). For not allowing more self-confident $\mathcal{P}$ to impose his maximally probable opinion, $\mathcal{Q}$ can have a larger confirmation bias.

---


[1] Genest C, Zidek JV (1986). Combining probability distributions: A critique and an annotated bibliography. Statistical Science 1: 114–135.

[2] Clemen RT, Winkler RL (1999). Combining probability distributions from experts in risk analysis. Risk Analysis 19: 187-203.

[3] Bernardo JM (1979). Expected information as expected utility. Annals of Statistics 7: 686-690.

[4] Alchourrón CE, Gärdenfors G, Makinsin D (1985). On the logic of theory change. J. Symb. Logic 50: 510-530.

[5] Keller AM, Winslett M (1985). On the use of extended relational model to handle changing incomplete information. IEEE Trans. Software Engineering SE-11: 620-633.

[6] Hogarth RM, Einhorn HJ (1992). Order effects in belief updating: The belief-adjustment model. Cognitive Psychology, 24: 1-55.

[7] Fink EL, Kaplowitz SA, Bauer CL (1983). Positional discrepancy, psychological discrepancy, and attitude change: Experimental tests of some mathematical models. Communication Monographs 50: 413-430.

[8] Bartlett MS (1945). Negative probability. Mathematical Proceedings of the Cambridge Philo-

sophical Society 41: 71-73.

[9] Allen EH (1976). Negative Probabilities and the Uses of Signed Probability Theory. Philosophy of Science 43: 53-70.

[10] Szekely GJ (2005). Half of a coin: negative probabilities. Wilmott Magazine 50: 66-68.

[11] Burgin M, Meissner G (2012). Negative probabilities in financial modeling. Wilmott Magazine 58: 6065.

[12] Marshall AW, Olkin I (1979). Inequalities: Theory of Majorization and its Applications. Academic Press, New York.

[13] Allahverdyan AE, Galstyan A (2011). Le Chatelier principle in replicator dynamics. Physical Review E 84: 041117

[14] Curtis JP, Smith FT (2008). Mathematical Models of Persuasion. American Conference on Applied Mathematics (MATH '08), Harvard, Massachusetts: 60-65.
